# Antibody Engineering for Pursuing a Healthier Future

**DOI:** 10.3389/fmicb.2017.00495

**Published:** 2017-03-28

**Authors:** Abdullah F. U. H. Saeed, Rongzhi Wang, Sumei Ling, Shihua Wang

**Affiliations:** Key Laboratory of Pathogenic Fungi and Mycotoxins of Fujian Province, Key Laboratory of Biopesticide and Chemical Biology of Education Ministry, and School of Life Sciences, Fujian Agriculture and Forestry UniversityFuzhou, China

**Keywords:** antibody engineering, hybridoma technology, antibody fragments, scFv, phage display technology, immunomodulation, immunology

## Abstract

Since the development of antibody-production techniques, a number of immunoglobulins have been developed on a large scale using conventional methods. Hybridoma technology opened a new horizon in the production of antibodies against target antigens of infectious pathogens, malignant diseases including autoimmune disorders, and numerous potent toxins. However, these clinical humanized or chimeric murine antibodies have several limitations and complexities. Therefore, to overcome these difficulties, recent advances in genetic engineering techniques and phage display technique have allowed the production of highly specific recombinant antibodies. These engineered antibodies have been constructed in the hunt for novel therapeutic drugs equipped with enhanced immunoprotective abilities, such as engaging immune effector functions, effective development of fusion proteins, efficient tumor and tissue penetration, and high-affinity antibodies directed against conserved targets. Advanced antibody engineering techniques have extensive applications in the fields of immunology, biotechnology, diagnostics, and therapeutic medicines. However, there is limited knowledge regarding dynamic antibody development approaches. Therefore, this review extends beyond our understanding of conventional polyclonal and monoclonal antibodies. Furthermore, recent advances in antibody engineering techniques together with antibody fragments, display technologies, immunomodulation, and broad applications of antibodies are discussed to enhance innovative antibody production in pursuit of a healthier future for humans.

## Introduction

In recent years, the development of polyclonal and monoclonal antibody by means of laboratory animals has become a vital approach to protect against a number of pathogenic contagions (Marasco and Sui, 2007). These immunoprotective molecules provide defense against transmissible diseases and can eliminate the infection. Their prophylactic and therapeutic protection ability was first discovered in the late nineteenth century by the passive transmission of antibodies from a diseased animal that provided immunity against diphtheria. Subsequently, immune sera from various herbivores and humans were obtained, pooled, and used as therapeutics. Since then, the management of infectious diseases such as diphtheria, tetanus, pneumococcal pneumonia, meningococcal meningitis, and toxin-mediated diseases has considerably improved patient survival (Casadevall, 1999).

Antibodies consist of two heavy chains [variable (V_H_), joining (J_H_), diversity (D), and constant (C) region] and two light chains [variable (V_H_), joining (J_H_), and constant (C) region], that are linked by non-covalent bonding and disulfide (s-s) bridges (Hamers-Casterman et al., 1993). Antibodies bind antigen with the help of a VHH fragment that can identify specific and unique conformational epitopes by the presence of its long complementary determining regions (CDR3). *Escherichia coli* expression systems are unique for the validation of the correct functioning of antibody fragments in the periplasmic space or cytoplasm. Conversely, periplasmic expression systems help V_H_ and V_L_ pairing by providing optimal conditions to allow the production of functional molecules (Sonoda et al., 2011).

Polyclonal antibodies contain large and diverse concentrations of different antibodies with unknown specificities. They are broadly used for the detection of different antigens in research and diagnostics. However, non-human polyclonal antibodies induce immune responses in humans that impede their clinical use such as treating snake bites (Wilde et al., 1996). Monoclonal antibodies have revolutionized scientific research. Production of these molecules is based on the fusion of antibody generating spleen cells from immunized mice, rats, or rabbits with immortal myeloma cell lines. These monoclonal antibodies are a highly specific class of biological reagents that facilitate enhanced clinical diagnostics in the medical arena. Subsequently, various antibodies are used clinically as prophylactic or therapeutic agents. The first monoclonal antibody developed by hybridoma technology was reported in 1975 and subsequently licensed in 1986 (Köhler and Milstein, 1975; Nelson, 2010). This development technique signifies a novel way to target specific mutations in nucleic acids and provide extensive expression in disease and other conditions (Nelson et al., 2010).

Antibody production was primarily dependent on animal immunization until the late 1980s by using experimental mice, rabbits and other related laboratory animals (Wang et al., 2010). The main difficulty in the production and application of monoclonal antibodies is the incompetent immune response to highly toxic or conserved antigens. Furthermore, most clinical antibodies are of human origin or are at least humanized in some aspect to avoid immunogenicity (Reichert, 2013). Therefore, transgenic mice and rabbits with human antibody genes have been developed to solve this immunogenicity problem but not the necessity of an effective immune response after immunization. Finally, to overcome this problem, human antibodies were generated *in vitro* by antibody engineering technologies such as phage display, construction of antibody fragments, immunomodulatory antibodies, and cell-free systems (Edwards and He, 2012).

Expression of recombinant antibodies *in vitro* experienced a boost with the advent of new molecular tools using various model organism such as yeast, bacteria etc., and new techniques for the selection of genetically engineered recombinant libraries using phage display technology. The phage display technique was first established by George P. Smith, when he validated the display of exogenous proteins on filamentous phage by fusing the peptide of interest to gene III of the phage. The first recombinant antibody fragments were constructed in bacteria 17 years ago (Roque et al., 2004). The goal of antibody production technology is to achieve high-titers of highly specific, and high-affinity antisera. Antigen preparation and animal immunizations are carried out following the guidelines of production techniques via hybridoma technology and recombinant technology (Smith, 1985). Moreover, therapeutic antibodies have been developed by modulation to the fragment crystallizable (Fc) receptor function and contribution of Fc glycan to immunoglobins, and the regulation of the antibody glycosylation in relation to immunoglobins-based therapeutics (Shade and Anthony, 2013).

Human diseases have been known for ages. The comfort of global travel and better interdependence have supplemented layers of intricacy to comprehend infectious diseases. These life threatening contagions effect human health in relation to unpredicted illnesses, deaths, and interfere many other normal life activities. Moreover, the diseases take a significant human toll as well as cause public fear (Morens and Fauci, 2013). To date, limited knowledge is available on extended aspects of the production of antibodies by hybridoma technology, antibody engineering techniques, construction of antibody fragments, display technologies, and their extended applications (Fauci and Morens, 2012). Therefore, to cope these health threats and limitations, extraordinary advances in hybridoma technology and antibody engineering techniques for the development of countermeasures (diagnostics, and treatment by therapeutic antibodies) have been discussed in the present review. Additionally, widespread antibody applications have been described in detail for pursuing a healthier future for humans, and to live a happy life.

## Polyclonal antibody

**Figure 1:**
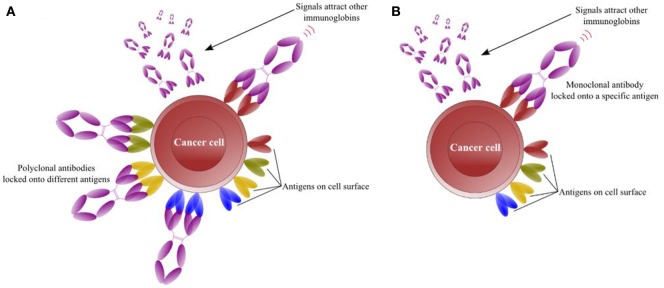
**Interaction of antibodies with numerous antigens present on the surface of target cell. (A)** Interaction of polyclonal antibodies with specific surface antigen activates B lymphocytes to divide and differentiate into plasma cell clones producing more antibodies that recognize antigens. **(B)** Interaction of monoclonal antibodies with specific surface antigen activates B lymphocytes to divide and differentiate into plasma cell clones that further recruit homogeneous and mono-specific antibodies.

Antigen interactions are essential for the normal functions of antibodies that are widely used in research or therapeutics. The antigen-specific and membrane-associated receptor antibody response is mediated by T and/or B cells. Consequently, upon binding with a suitable antigen, B lymphocytes are induced to proliferate, and divide by a number of activating signals, thus increasing the numbers of B cells. These B cells are then differentiated into specific antibody producing plasma cell clones that recognize specific antigen epitopes via the antigen receptor. B cells are activated after recognizing their specific antigen (Figure 1A; Andersen et al., 2006). Some antigens are highly multifarious and exhibit abundant epitopes recognized by several lymphocytes. Consequently, lymphocytes multiply and differentiate by activation of these multifarious antigens into plasma cells that produce polyclonal antibody responses (McCullough and Summerfield, 2005).

Polyclonal antibodies (pAbs) can be produced rapidly in several months (compared to monoclonal antibodies), with low cost, low technical skill, and these are stable over a broad range of pH and salt concentrations. In addition, they have applications as therapeutic immunoglobulins (Lipman et al., 2005). PAbs recognize multiple linear epitopes with minimal conformational changes and contain numerous antibodies of varying affinities, which are useful for the immunoprecipitation of composite antigens to form a large precipitating lattice. Mice are used often to produce pAbs because of their small size and blood volume. As an alternative, pAbs are produced as ascites in mice. Moreover, these antibodies have superior specificity compared with monoclonal antibodies because they are generated by a large number of B-cell clones each producing antibodies to a specific epitope (Hudson et al., 2012; Zhuang et al., 2014).

## Monoclonal antibody

Monoclonal antibodies (mAbs) are clinically significant homogeneous and mono-specific scientific biomolecules produced from hybridoma cells by hybridoma technology (Zhang, 2012). mAbs arise from single cell clone compared to multiple cell clones for pAbs (Figure 1B; Andersen et al., 2006). Since their discovery, these molecules have been used as research tools and have revolutionized the fields of biotechnology, immunology, diagnostics, and medicine. The technology was described for the first time by Köhler and Milstein (1975) in the mid-1970s in the journal Nature, and they were later awarded the Nobel Prize (Saeed and Awan, 2016).

Currently, mAb products approved by the US Food and Drug Administration (FDA) are increasing worldwide i.e., about four new products per year. Currently, 47 mAb products in the US, Europe and global markets have been approved for the treatment of a variety of diseases (Table 1; Ecker et al., 2015). At the current rate, about 70 mAb products will be on the market by 2020, and collective global trade will be approximately $125 billion (Ecker et al., 2015). Improvements in hybridoma technology are based on research demand, cost effectiveness, human labor, and reduced development time. Similarly, the production of mAbs requires multiple phases, long duration, and high cost. Currently, mAbs have been produced against a number of mycotoxins such as fumonisin B1 (Yuan et al., 2012; Ling et al., 2014, 2015b), citreoviridin (Jin et al., 2014), marine toxins (Saeed and Wang, 2016), and other exo- and endo-antigens. Similarly, mAbs against transmembrane enzymes have been produced (Yuan et al., 2012).

**Table 1:** **Monoclonal antibody products in the US, Europe, and global markets approved for diseases**.

**Brand name**	**Company reporting US sales**	**Company reporting EU sales**	**Year of approval**	**Treatment**
AlprolIX (Factor IX Fc fusion protein)	Biogen Idec	Sobi and Biogen Idec	2014	Hemophilia B
Cyramza (ramucirumab)	Eli Lilly and Co.	Eli Lilly and Co.	2014	Gastric cancer and non-small cell lung cancer
Eloctate (Factor VIII Fc fusion protein)	Biogen Idec	Sobi and Biogen Idec	2014	Anti-hemophilic Factor
Entyvio (vedolizumab)	Takeda Pharmaceutical Co.	Takeda Pharmaceutical Co.	2014	Ulcerative colitis (UC)/Crohn's disease (CD)
Keytruda (pembrolizumab)	Merck & Co.	Merck & Co.	2014	Melanoma
Sylvant (siltuximab)	Johnson & Johnson	Johnson & Johnson	2014	Multicentric Castleman's Disease (MCD)
Inflectra (infliximab [biosimilar])	N/A	Hospira	2013	Tumor necrosis
Kadcyla (ado-trastuzumab emtansine)	Roche	Roche	2013	Metastatic breast cancer
Lemtrada (alemtuzumab)	N/A	Sanofi	2013	Relapsing form of multiple sclerosis (MS)
Gazyva (obinutuzumab)	Roche	Roche	2013	Chronic lymphocytic leukemia (CLL)
Remsima (infliximab [biosimilar])	N/A	Celltrion	2013	Rheumatoid arthritis, psoriatic arthritis, ulcerative colitis, Crohn's disease, ankylosing spondylitis, and plaque psoriasis
Perjeta (pertuzumab)	Roche	Roche	2012	Human epidermal growth factor receptor 2 (HER2)/neu-positive) metastatic breast cancer
Abthrax (raxibacumab)	GlaxoSmithKline	GlaxoSmithKline	2012	Inhalational anthrax
Prolia (denosumab)	Amgen	GlaxoSmithKline	2011	Osteoporosis
Adcetris (brentuximab vedotin)	Seattle Genetics	Takeda Pharmaceutical Co.	2011	Hodgkin lymphoma
Benlysta (belimumab)	GlaxoSmithKline	GlaxoSmithKline	2011	Systemic lupus erythematosus (SLE/lupus)
Eylea (aflibercept)	Regeneron Pharmaceuticals	Bayer Healthcare Pharmaceuticals	2011	Macular degeneration
Nulojix (belatacept)	Bristol-Myers Squibb	Bristol-Myers Squibb	2011	Prevention of transplant rejection
Xgeva (denosumab)	Amgen	Amgen	2010	Prevention of bone fractures and other skeletal bone tumor conditions
Arzerra (ofatumumab)	GlaxoSmithKline	GlaxoSmithKline	2009	Chronic lymphocytic leukemia (CLL)
Ilaris (canakinumab)	Novartis Pharmaceuticals	Novartis Pharmaceuticals	2009	Systemic Juvenile Idiopathic Arthritis (SJIA)
Actemra (tocilizumab)	Roche	Roche	2009	Moderate to severe rheumatoid arthritis
Simponi Aria (golimumab)	Johnson & Johnson	Merck & Co.	2009	Rheumatoid arthritis
Stelara (ustekinumab)	Johnson & Johnson	Johnson & Johnson	2009	Plaque psoriasis, psoriatic arthritis and Crohn's disease
Removab (catumaxomab)	N/A	NeoPharm Group	2009	Malignant ascites
Arcalyst (rilonacept)	Regeneron Pharmaceuticals	Regeneron Pharmaceuticals	2008	Familial Cold Auto-inflammatory Syndrome/Muckle-Wells Syndrome
Cimzia (certolizumab pegol)	UCB	UCB	2008	Rheumatoid Arthritis
Nplate (romiplostim)	Amgen	Amgen	2008	Low blood platelet counts
Soliris (eculizumab)	Alexion Pharmaceuticals	Alexion Pharmaceuticals	2007	Paroxysmal nocturnal hemoglobinuria (PNH), and Atypical hemolytic uremic syndrome (aHUS)
Lucentis (ranibizumab)	Roche	Novartis Pharmaceuticals	2006	Macular degeneration
Vectibix (panitumumab)	Amgen	Amgen	2006	Metastatic colorectal cancer
Orencia (abatacept)	Bristol-Myers Squibb	Bristol-Myers Squibb	2005	Rheumatoid arthritis
Avastin (bevacizumab)	Roche	Roche	2004	Various cancers and eye disease
Tysabri (natalizumab)	Biogen Idec	Biogen Idec	2004	MS and Crohn's disease
Erbitux (cetuximab)	Bristol-Myers Squibb	Merck KGaA	2004	Metastatic colorectal cancer, metastatic non-small cell lung cancer, head and neck cancer
Humira (adalimumab)	AbbVie	AbbVie	2002	Rheumatoid arthritis, juvenile idiopathic arthritis, psoriatic arthritis, ankylosing spondylitis, plaque psoriasis, and hidradenitis suppurativa
Enbrel (etanercept)	Amgen	Pfizer	1998	Rheumatoid arthritis
Herceptin (trastuzumab)	Roche	Roche	1998	Breast cancer/stomach cancer
Remicade (infliximab)	Johnson & Johnson	Merck & Co.	1998	Crohn's Disease
Simulect (basiliximab)	Novartis Pharmaceuticals	Novartis Pharmaceuticals	1998	Prevention of organ rejection
Synagis (palivizumab)	AstraZeneca	Abbvie	1998	Lung disease caused by respiratory syncytial virus (RSV)
Rituxan (rituximab)	Roche	Roche	1997	Non-Hodgkin's lymphoma or chronic lymphocytic leukemia
ReoPro (abciximab)	Lilly	Lilly	1994	Prevention of blood clots

### Method of antibody production

**Figure 2:**
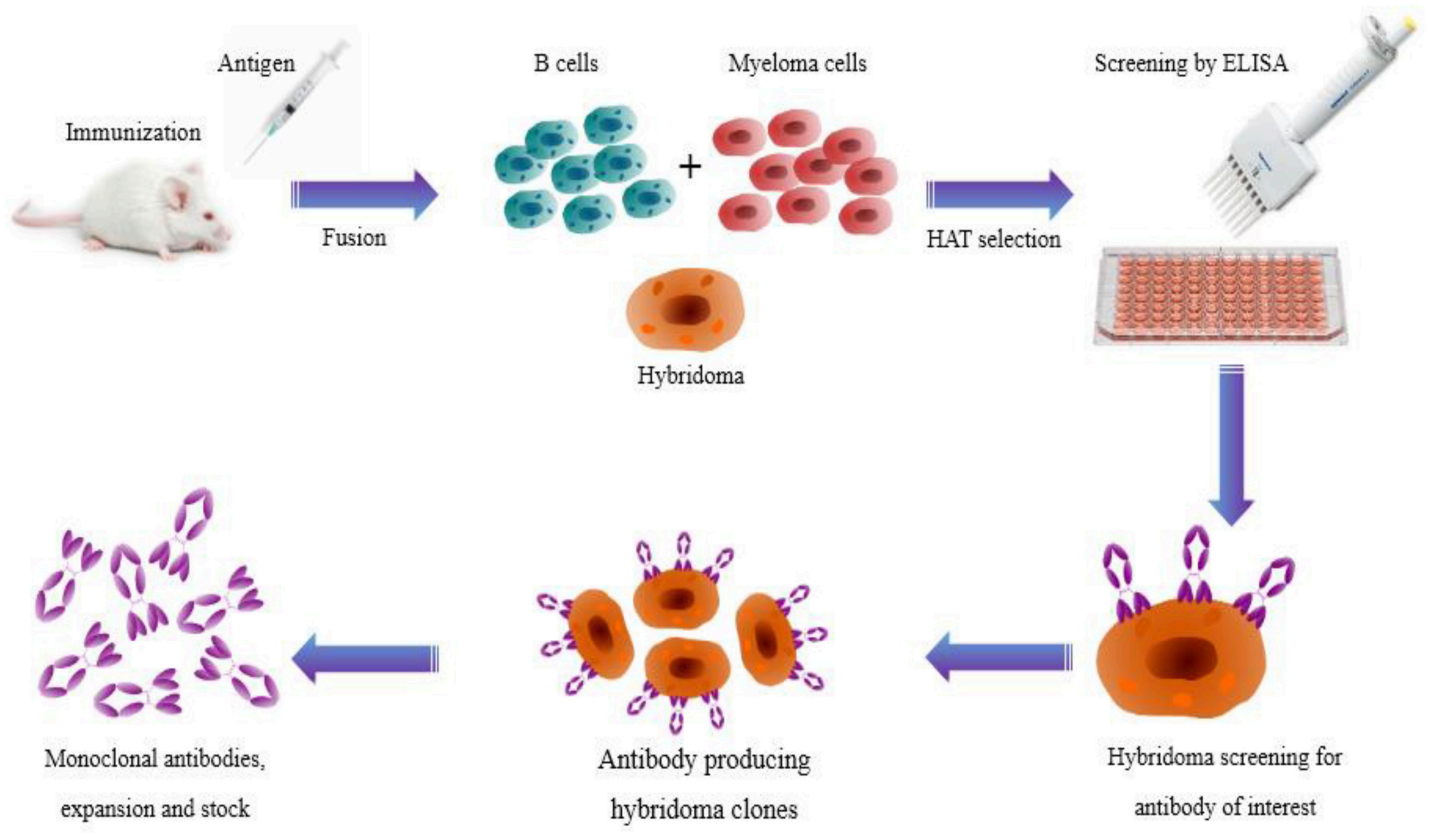
**Illustration showing the production route of hybridoma technology**. Monoclonal antibodies are generated by immunizing laboratory animals with a target antigen. B cells and myeloma cells are fused and then selected in HAT medium. Finally, hybridoma cells producing the desired antibodies are screened.

Hybridoma technology has been a significant and essential platform for producing high-quality mAbs (Zhang, 2012). It permits generation of therapeutic antibodies in a native form. However, technical difficulties in hybridoma production have updated the mainstream antibody production into new ways like display and transgenic mice techniques. Nevertheless, hybridoma technology is a classical and established route of generating specific antibodies all around the globe (Glukhova et al., 2016). The technology begins with immunization of test animals with an antigen of interest and serum antibody titer is determined by enzyme linked immunosorbent assay (ELISA). Subsequently, the spleen is aseptically removed and splenocytes are fused with myeloma cells to produce hybridoma cells. Hybridoma cells are then cultured in 96-well plates in the presence of hypoxanthine-aminopterin-thymidine (HAT) selection medium for high throughput screening. Later, hybridoma cells producing desired antibodies are screened by conventional ELISA and novel nanoparticle-probed immunoassay (colloidal gold or silver nanoparticles; Figure 2). Cell culture systems *in vitro* with specific mAb cell lines were then subjected to mass generation by media selection, shaker flasks, and bench-scale bioreactors (Ling et al., 2014).

The optimal conditions such as temperature, percent carbon dioxide, and humidity for cell cultures should be determined (Sen and Roychoudhury, 2013), and then transferred to a pilot scale for scalability and toxicology studies. In addition, clinical materials should be produced on a large scale under the existing good manufacturing practice (cGMP) regulations. After production on a small scale, products that are already cultured in a laboratory are then transferred to pilot scale commercialization. The process then performs characterization, scaling, technology transfer, and validation. Commercial cell culture for the production of a biological product is completed by pilot scale laboratory methods (Li et al., 2010). Recently, commercialization is initiated by process characterization, scale-up, technology transfer, and validation of the manufacturing process (Li et al., 2006).

ELISA is enzyme-based colorimetric assay, requires large sample volumes, several incubation steps and has low detection sensitivity (Tang and Hewlett, 2010). Conversely, nanotechnology and nanoparticles (NPs) use nanomaterials with length scale of 1–100 nanometers (nm). Nanomaterials have unique biological properties such as small size, large surface-to-volume ratio, sharp melting temperature, magnetic properties, unusual target binding properties, and size based multi-coloring (Qi and Wang, 2004). NPs such as gold particles have been used to improve assay sensitivity and specificity of antibodies, low limit of detection (LOD), dose response over 10,000-fold and the detection sensitivity by 1,000-fold compared to ELISA (Tang and Hewlett, 2010).

Similarly, an essential factor of NPs selection is that nanometer-sized (colloidal gold or silver) particles can be conjugated with targeting ligands, including mAbs, peptides, or small molecules with functional and structural properties that are not available from either discrete molecules or bulk materials. Nanoparticles are probed with mAbs that can be used to target any antigen of interest (Ling et al., 2015a). The use of colloidal gold is a rapid, less time consuming, and cost effective technique. This technology has been used in the development of immunochromatic strip assays based on mAb. The technique has widespread applications for the detection of a number of antigens (exogenous antigens, endogenous antigens, autoantigens, neoantigens, viral antigens, and tumor antigens) with high specificity and binding affinity (Schumacher and Schreiber, 2015).

### Antigen preparation

Antigen preparation including quality and quantity is essential for the production of antibodies. The purification of antigens is difficult and time consuming but antigen purity is vital for adequate immune responses (Leenaars and Hendriksen, 2005). Insignificant impurities (<1%) can be immunodominant in the case of various microbial antigens. They show non-specific activity against the antigens of interest and specific activity against impurities. Higher concentrations of specific antibodies are obtained after purification and the exclusion of impurities by extensive absorption procedures such as antibody affinity chromatography (Leenaars et al., 1997; Mazzer et al., 2015).

Before scheduling immunization, antigen contamination should be considered by the researcher. The antigen and diluents should be free of endotoxins such as lipopolysaccharide, or chemical residues that have been utilized to neutralize the microorganism. Additionally, the pH must be adjusted to prevent undesirable effects in the animal to be immunized (Hendriksen and Hau, 2003). Moreover, sterile working conditions, antigen concentration, animal preparation, and injection quality must be confirmed. These conditions are necessary to avoid suppression, sensitization, tolerance, or other superfluous immunomodulation and to induce effective immune responses. The required antigen concentration (μg to mg) is based on the intrinsic properties of the antigen. Usual doses of antigen conjugated with Freund's adjuvant for rabbits are in the range of 50–1,000 μg; for mice, 10–200 μg; and for goats and sheep, 250–5,000 μg (Leenaars et al., 1997). The inherent properties of antigen quantity include purification, animals to be immunized, type, and quality of the adjuvant (to elicit high-titer serum responses), route and the immunization (injection) frequency (Hanly et al., 1995).

### Immunization

Antibody production involves the immunization or injection of laboratory animals with an immunogenic protein and test sampling of antiserum. The immunization is performed in specific animal species and the adjuvant is processed to form an immunogenic emulsion that is insoluble in water (Leenaars and Hendriksen, 2005). Conjugation of smaller or less immunogenic antigens i.e., haptens (numerous myco- and marine toxins) to carrier proteins is essential before animal injection. After immunization, the animal is monitored daily or three times a week for side effects (clinical and pathological examination). Severe pathological changes such as tissue reactivity, infection and anaphylactic reactions in case of booster injections have been reported in the absence of visual clinical or behavioral changes (Leenaars et al., 1997).

The immunization route is based on the choice of animal species, adjuvant, concentration and quality parameters of the antigen (Apostolico Jde et al., 2016). Suitable immunization routes include subcutaneous (s.c.), intradermal (i.d.), intramuscular (i.m.), intraperitoneal (i.p.), and intravenous (i.v.). Blood sample handling should not interfere with the immunization site to avoid pain and distress to the animal while taking blood sample (Leenaars et al., 1997). The i.v. route for water-in-oil emulsions such as Freund's complete and incomplete adjuvants (FCA and FIA) may be fatal as embolisms caused by large particulates or viscous gel adjuvants (e.g., aluminum salts) can occur (Hanly et al., 1995). The s.c. administration of FCA is immunologically effective (0.1 mL in mice and 0.25 mL in rabbits) with some reports of pathological changes. The i.p. administration of FCA is not suggested for antibody development because it induces infection, peritonitis, and social variations. There is high risk of anaphylactic shock for booster injections by the i.v. or i.p. route (Leenaars et al., 1997).

The volume of the immunogenic mixture depends on the quality of the antigen and the degree of the lesions formed. Therefore, smaller volumes of mixtures are injected to induce antibody responses except when increased concentrations of antigen are required (Leenaars and Hendriksen, 2005). The immunization volume also depends on the animal species, route, and chemistry of the injection mixture. The volume of FCA has lethal effects on animal physiology and the extent of lesions produced is associated with high volumes of FCA (Ramos-Vara and Miller, 2014).

Booster injections have a significant effect on the outcomes of immunization and induction of B memory cells. Class switching of B cells depends on the time interval between two consecutive injections. A small volume of antigen can be used for a booster injection without adjuvant (Leenaars et al., 1997). A booster injection is used when the antibody titer has reached a plateau or begins to decline. Antibody titers typically peak at 2–3 weeks after immunization in the absence of the depot-forming adjuvant used in the first immunization. A booster injection is used after 4 weeks in the presence of depot-forming adjuvant. FCA should be used in the first s.c. injection to avoid subsequent (*Mycobacteria* proteins) severe tissue reactions (Hendriksen and Hau, 2003).

### Hybridoma screening

Hybridoma technology is essential for the production of high-quality mAbs as well as research and diagnostic reagents, and it is currently the most rapidly growing class of therapeutics (Hnasko and Stanker, 2015). MAbs can be effectively screened by a number of techniques such as ELISA, phage display, and other related technologies. Screening identifies and picks only specific antibody producing hybridomas. In hybridoma technology, laboratory animals are immunized with an antigen of interest and specific antibody-producing B cells are isolated from the spleen and then fused with immortal myeloma cells (such as sp2/0; Bradbury et al., 2011). Subsequently, expansions of clonal populations are produced from serially diluted sub-cloned individual hybridoma cells in a microtiter plate. Next, the hybridoma supernatant is checked by ELISA or other related immunoassays for desired antibody activity (El Debs et al., 2012).

After screening, hybridoma cells are expanded in bigger wells or in culture flasks to maintain the hybridoma and provide sufficient cells for cryopreservation and supernatants for later investigations (Nelson et al., 2010; El Debs et al., 2012). However, this technique restricts the number of clones that can be screened to no more than a few thousand. Consequently, insufficient numbers of cells remain available for clonal expansion and cell immortalization. Therefore, improved techniques such as antigen-based microarrays have been used for the screening of >10^5^ clones in <12 h, or lithographically fabricated microwells for individual cell compartmentalization. Nevertheless, these techniques only screen for binding activity and do not allow functional assays (El Debs et al., 2012).

### Characterization of monoclonal antibodies

MAb characterization is based on a determination of its physicochemical and immunochemical character, heterogeneity, purity, impurities, biological activity, potency, and concentration (Berkowitz et al., 2012). For physicochemical properties, the isotype (class, subclass, light chain composition) and primary structure of the mAb are determined. Immunological properties include binding assay, affinity, avidity, immunoreactivity (cross-reactivity with other structurally homologous proteins), analysis of CDR, epitope characterization, and determination of effector functions (CHMP, 2008).

Heterogeneity (including chromatographic and electrophoretic methods) and quantity are determined for the characterization of mAb purity, impurity, contaminants, and concentration. Specificity of mAbs is also checked. Peptide map, anti-idiotype immunoassay, potency, and other appropriate methods are used to determine its identity and biological activity (CHMP, 2008).

### Applications of monoclonal antibodies

MAb-based products exhibit superior specificity for a particular antigen. This characteristic feature of the immunoglobins makes them an ideal tool for many applications including disease diagnosis and therapy (Table 2; Modjtahedi et al., 2012; Redman et al., 2015; Steplewski et al., 2015). Diagnostic applications include biochemical analysis and imaging. It involves a number of immunoassays for the detection of hormonal, tissue, and cell products. Imaging is carried out using radiolabeled mAbs for diagnostics of infectious diseases. Therapeutic mAbs have a wide range of applications. They are used in the treatment of cancer, transplantation of bone marrow and organs, autoimmune diseases, cardiovascular diseases, and various infectious diseases. Therapy can be carried out by direct use of mAbs as therapeutic agents and as targeting agents respectively (Modjtahedi et al., 2012).

**Table 2:** **Summary of various applications of monoclonal antibodies**.

**Application**	**Features**	**Diagnosis, treatment, and gains**
**DIAGNOSIS**		
Biochemical analysis	Diagnostic tests are regularly used in radioimmunoassay (RIA) and ELISA in the laboratory to quantify the circulating concentrations of hormones and several other tissue and cell products.	Pregnancy: human chorionic gonadotropin; cancers: colorectal cancer, prostate cancer, tumor markers; hormonal disorders: thyroxine, triiodothyronine, thyroid; infectious diseases, sexually transmitted diseases (STDs) include *Neisseria gonorrhoeae*, herpes simplex virus.
Diagnostic imaging	The technique is also called immunoscintigraphy. Radiolabeled—mAbs are used in the diagnostic imaging of the diseases. The radioisotopes commonly used for labeling mAbs are iodine—131 and technetium—99. Imaging tool include single photon emission computed tomography (SPECT) cameras.	Myocardial infarction: myocardial necrosis; deep vein thrombosis: thrombus in thigh, pelvis, calf, knee; atherosclerosis: coronary and peripheral arteries; immunohistopathology of cancers: colon, stomach, pancreas, liver, germ cells of testes, choriocarcinoma, prostate cancer, melanoma; hematopoietic malignancies: hematopoietic stem cells malignancy; bacterial infections.
**THERAPEUTIC APPLICATIONS**		
Direct therapeutic agents	Monoclonal antibodies can be directly used for augmenting the immune function of the host triggering minimal toxicity to the target tissues or the host.	Opsonization and phagocytosis of pathogenic organisms: hepatitis B-virus, *E. coli*, haemophilus influenza, *streptococcus* sp. pseudomonas sp; cancer treatment: ADCC, CDC, phagocytosis of cancer cells, colorectal cancer, lymphoma, melanoma; immunosuppression of organ transplantation; treatment of AIDS; treatment of autoimmune diseases: rheumatoid arthritis, MS.
Targeting agents in therapy	Toxins, drugs, radioisotopes etc., can be attached or conjugated to the tissue-specific mAbs and carried to target tissues for efficient action.	Immunotoxins: diphtheria toxin, pseudomonas exotoxin, toxins used for cancer treatment; drug delivery: antibody-directed enzyme pro-drug therapy (ADEPT), liposomes coupled mAb drug delivery; dissolution of blood clots: thrombus in coronary or cerebral artery; immunotherapy (RAIT): yttrium-90, indium-111.
Protein Purification	MAbs are produced against protein of interest and conveniently used for the purification of that particular protein.	In a single step, it is likely to reach more than 5,000-fold purification of interferon-α2.
**MISCELLANEOUS APPLICATIONS**		
Catalytic mAbs (ABZYMES)	The antibody enzymes, appropriately regarded as abzymes, are the catalytic antibodies. Hapten-carrier complex is formed that resembles the transition state of an ester undergoing hydrolysis. This hapten conjugate is used to produce anti-hapten mAbs.	Widespread applications include splicing of peptides and deoxyribonucleic acids, dissolution of blood clots, and killing of viruses.
Autoantibody fingerprinting	Recently, a new class of individual specific (IS) autoantibodies have been documented in recent years. These IS-autoantibodies are developed after birth and extend maximum in number by 2 years, and then stay persistent afterwards.	Autoantibodies collected from blood, saliva, semen and tears are used for detection, and identification of individuals especially for forensic sciences.

### Computational and bioinformatics tools for antibody selection

Computational and bioinformatics approaches play an essential role for antibody selection and epitope prediction. It is an interdisciplinary science and the term can be defined as “the application of computer tools to handle biological information.” Several computational and bioinformatics tools for prediction of antibody binders include RANKPEP, nHLAPred, NetMHC, kernel-based inter-allele peptide binding prediction system (KISS) with support vector machine (SVM; Bhasin and Raghava, 2007; Lundegaard et al., 2008). Moreover, these tools use databases that contain known epitopes from SYFPEITHI, MHCBN, LANL, and IEDB for protein epitope prediction (Soria-Guerra et al., 2015).

Furthermore, other tools include position specific scoring matrices (PSSM) used for sequence alignment, IEDB analysis resource database uses NetMHCpan for peptide affinity, quantitative matrices (QM), whole antigen processing pathway (WAPP), Matthews correlation coefficient (MCC), and spatial epitope prediction of protein antigens (SEPPA) (Soria-Guerra et al., 2015). Recently, a simulation tool C-ImmSim was developed for the study of a number of different immunological processes. The processes include simulations of immune response by representing pathogens, as well as lymphocytes receptors, amino acid sequences and T and B cell epitope prediction (Rapin et al., 2010).

## Antibody engineering

The first fully human mAb was developed over 25 years ago by phage display and a selection of antigen-specific binders from blood lymphocyte libraries (Gavilondo and Larrick, 2000). This technique employed transgenic animals such as mice and rabbits with integrated human immunoglobulin (Ig) loci. Germline-configured chimeric constructs confirmed that human, mouse, and all mammalian Ig loci function in very similar ways (Neuberger and Bruggemann, 1997). Antibody development has progressed from hybridoma technology to a recombinant deoxyribonucleic acid (DNA) approach. In the last few years, a number of engineered antibody drugs have been approved or investigated in phase II or III clinical trials (Table 3; Nelson et al., 2010; Dantas-Barbosa et al., 2012; Nixon et al., 2014).

**Table 3:** **Engineered antibody drugs in phase II, phase III clinical trials or approved**.

**International drug code**	**Company/sponsor**	**Type/source**	**Target antigen**	**Progress status**	**Disorder(s)**	**Administration route**
Nivolumab (OPDIVO)	Bristol-Myers Squibb	Human IgG4 monoclonal antibody	Programmed cell death protein 1 (PD-1)	Approved (2017)	Locally advanced or metastatic urothelial carcinoma	IV
Necitumumab (Portrazza)	Eli Lilly and Company	Humanized IgG1 monoclonal antibody	Epidermal Growth Factor Receptor (EGFR)	Approved (2015)	Cancer (NSCLC)	IV
Ramucirumab (Cyramza)	Eli Lilly and Company	Human IgG1 monoclonal antibody	Vascular endothelial growth factor receptor 2 (VEGFR2)	Approved (2015)	Cancer (NSCLC, breast, metastatic gastric adenocarcinoma)	IV
Raxibacumab (ABthrax)	CAT technology and Human Genome Sciences- GlaxoSmithKline	Recombinant Human IgG1λ Monoclonal Antibody	Protective antigen (PA) component of anthrax (*Bacillus anthracis*)	Approved (2012)	Prophylaxis, anthrax	Oral or IV
Ipilimumab (Yervoy)	Bristol-Myers Squibb	Human IgG1κ monoclonal antibody	Human cytotoxic T-lymphocyte antigen 4 (CTLA-4)	Approved (2011)	Melanoma, Metastatic	IV
Belimumab (Benlysta)	GlaxoSmithKline	Recombinant human IgG1λ monoclonal antibody	B-lymphocyte stimulator (BLyS)	Approved (2011)	Autoantibody-positive, systemic lupus	IV
Ecallantide (Kalbitor)	Dyax Corp.	human IgG1 monoclonal antibody	Plasma kallikrein	Approved (2009)	Hereditary angioedema	SI
Romiplostim (Nplate)	Amgen Inc.	Peptide-Fc fusions or peptibody	Thrombopoietin receptor (TPOR)	Approved (2008)	Immune thrombocytopenic purpura	SI
Ranibizumab (Lucentis)	Genentech	Recombinant humanized IgG1κ monoclonal antibody fragment	Vascular endothelial growth factor A (VEGF-A)	Approved (2006)	Neovascular (wet) age-related macular degeneration	Intravitreal injection
Adalimumab (Humira)	Abbott Laboratories	Recombinant human IgG1 monoclonal antibody	Tumor necrosis factor-α (TNF)	Approved (2002)	Rheumatoid arthritis, juvenile idiopathic arthritis, psoriatic arthritis, ankylosing spondylitis, crohn's disease, ulcerative colitis, plaque psoriasis	SI
Solofuse (Xyntha)	Wyeth Pharmaceuticals/ Pfizer	Recombinant factor VIII product	Factor VIII	Approved (2008)	Hemophilia A	IV
Guselkumab (CNTO 1959)	MorphoSys/Janssen Biotech	Human IgG1 monoclonal antibody	Interleukin 23 (IL-23p19)	Phase III	Psoriasis	IV or SI
Gantenerumab	Roche	Human IgG1 monoclonal antibody	Beta-amyloid	Phase III	Alzheimer	IV or SI
Trebananib (AMG 386)	Amgen Inc.	Angiopoietin 1/2-neutralizing peptibody	Angiopoietin 1 and 2 neutralizing peptibody (Ang1/2)	Phase III	Cancer (ovarian, peritoneal, fallopian tube)	IV
Foravirumab (CL-184)	Sanofi/Crucell	Human IgG1κ monoclonal antibody	Rabies virus glycoprotein	Phase III	Prophylaxis, inhaled anthrax	IV
Bimagrumab (BYM338)	Novartis Pharmaceuticals	Human IgG1λ monoclonal antibody	Activin receptor IIB (ActRIIB)	Phase IIb/III	Pathological muscle loss and weakness	IV
ZMapp	Mapp Biopharmaceutical, Inc.	Chimeric monoclonal antibody cocktail of MB-003, ZMab, c2G4 and c4G7	Ebola virus protein sGP	Phase II	Ebola virus disease (EVD)	IV
GRN1005	Angiochem Inc.	Peptide-drug conjugate	Lipoprotein receptor protein-1	Phase II	Non-small Cell Lung Cancer (NSCLC) With Brain Metastases	IV
Ganitumab (AMG 479)	Amgen Inc.	Human IgG1 monoclonal antibody	Insulin-like growth factor receptor (IGF-1R)	Phase II	Cancer (pancreatic, colorectal breast, NSCLC)	IV
Cixutumumab (IMC-A12)	Eli Lilly and Co.	Human IgG1 monoclonal antibody	Insulin-like growth factor-1 receptor (IGF-1R)	Phase II	Cancer (NSCLC, metastatic melanoma of the eye, liver)	IV
MM-121	Merrimack Pharmaceutical partner with Sanofi	Human IgG2 monoclonal antibody	ErbB3 gene	Phase II	Cancer (advanced ovarian, hormone sensitive breast cancer, NSCLC, and HER2 negative neoadjuvant breast cancer)	IV
BIIB 033	Biogen Idec	Human aglycosyl IgG1 monoclonal antibody	Leucine-rich repeat and immunoglobulin-like domain containing, Nogo receptor-interacting protein (LINGO 1)	Phase II	Acute optic neuritis, MS	IV
Mapatumumab	GSK company	Human IgG1 monoclonal antibody	TNF-related apoptosis-inducing ligand receptor 4 (TRAIL-4)	Phase II	Cancer (NSCLC, non-Hodgkin lymphoma, liver, cervical)	IV
Tralokinumab (CAT-354)	AstraZeneca	Human IgG4 monoclonal antibody	Interleukin-13	Phase IIb	Ulcerative colitis, pulmonary fibrosis, asthma	SI
Mavrilimumab (CAM-3001)	MedImmune	Human IgG4 monoclonal antibody	Granulocyte macrophage-colony stimulating factor receptor (GM-CSF)	Phase IIb	Rheumatoid arthritis	IV
Bertilimumab	Immune Pharmaceuticals	Human IgG4 monoclonal antibody	Eotaxin-1 (CCL-11 gene)	Phase II	Ulcerative colitis	IV
GSK3196165 (MOR103)	MorphoSys/ GSK company	Recombinant Human IgG1 monoclonal antibody	Granulocyte macrophage colony-stimulating factor (GM-CSF)	Phase II	Inflammatory diseases (rheumatoid arthritis)	IV
BHQ880	Novartis Pharmaceuticals	Human IgG1 monoclonal antibody	Dickkopf-1 (DKK1 gene)	Phase II	Multiple myeloma	IV
Carlumab (CNTO 888)	Centocor Research & Development, Inc.	Human IgG1κ monoclonal antibody	Monocyte Chemoattractant Protein-1 (MCP-1), CCL-2 gene	Phase II	Prostate cancer	IV
MLDL1278A (BI-204)	Genentech/ Bioinvent	Recombinant Human IgG1 antibody	Oxidized low-density lipoprotein (LDL)	Phase II	Stable atherosclerotic vascular disease	IV or SC
Adecatumumab (MT201)	Amgen Inc.	Recombinant Human IgG1 monoclonal antibody	Epithelial cell adhesion molecule (EpCAM)	Phase II	Colorectal cancer (CRC), liver and breast metastases	IV
Fresolimumab (GC-1008)	Sanofi/Genzyme	Human IgG4 monoclonal antibody/immunomodulator	Transforming growth factor-beta (TGF-β) 1, 2, and 3	Phase II	Primary brain tumors, primary focal segmental glomerulosclerosis, diopathic pulmonary fibrosis, cancer	IV
BI-505	BioInvent	Human IgG1 monoclonal antibody	Intercellular Adhesion Molecule 1 (ICAM-1) or Cluster of Differentiation 54 (CD54)	Phase II	Cancer (multiple myeloma)	IV

*NSCLC, non-small cell lung cancer; IV, intravenous; SI, subcutaneous injection; IM-intramuscular injection*.

MAb immunoglobulins (IgG) are the starting material for the generation of smaller antibody fragments in lymphoid or non-lymphoid cells (Klimka et al., 2000). Traditional hybridoma technology has several limitations such as being exclusively murine based, time consuming, and exhibiting low-affinity in conventional assays. Therefore, antibody engineering, display system, and immunomodulation methods are now used to produce efficient therapeutic antibodies (Klimka et al., 2000).

The first study of recombinant antibodies in bacteria was difficult because of interference from disoriented proteins in the bacterial cytoplasm. A new antibody expression technique was developed to produce smaller antibody molecules (Fab or Fv fragments; Okamoto et al., 2004) where numerous types of vectors (phagemid) are used with competent *E. coli*. This technique involves the expression of antibody fragments for recombinant antibody construction. In this way, a number of genetically engineered antibodies have been constructed, such as Fab fragments, Fv fragments, single-chain variable fragments, bivalent antibodies, and bispecific antibodies (Little et al., 2000).

### Antibody fragment display

**Figure 3:**
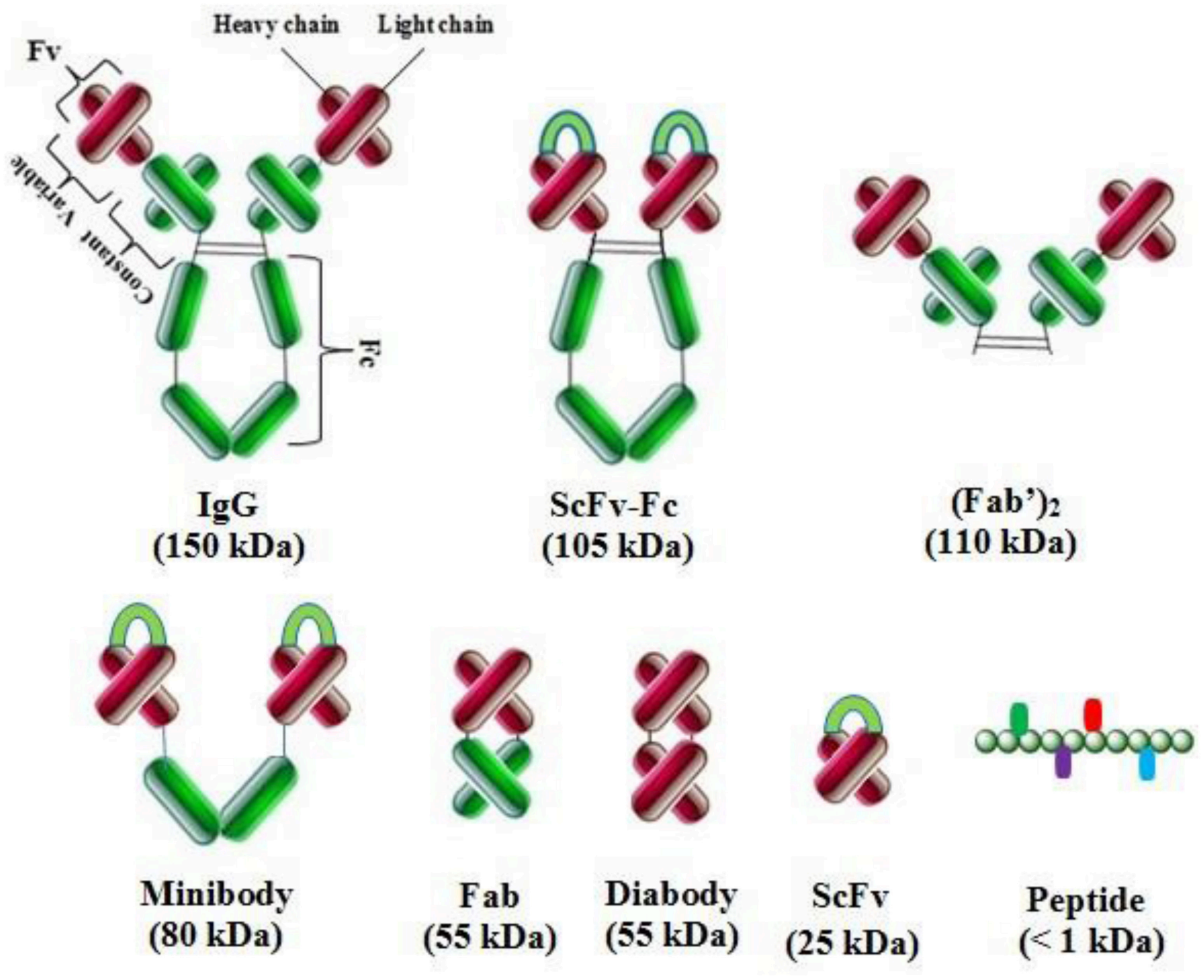
**Complete antibody and various types of antibody fragments**. These fragments are constructed by antibody engineering techniques for enhanced therapeutic applications.

Different antibody fragments are used in phage display technology: scFv (single chain fragment variable), Fv (fragment variable), Fab (antigen binding fragment) and their derivatives, V-gene domain, bispecific or bivalent antibodies, and other oligomers (Figure 3; Nelson, 2010). Fab fragments are the linkage of V_H_–C_H_ and V_L_–C_L_ by disulfide bridges, and radiolabeled Fabs are used in tumor imaging. Fv is used for the construction of V_L_ and V_H_ domains or their modifications such as scFv, which is the most popular fragment. A (Gly4Ser)3 linker is used for the stabilization of V_L_–V_H_ and proper antigen binding site formation without the loss of antibody affinity. Chelating recombinant antibodies (CRAbs) are scFv segments with a high binding affinity. These constructs consist of two scFv fragments specific to the identical antigen and adjacent epitopes. These fragments are connected by a short linker (up to 10 amino acids) for the dimerization and formation of diabodies (Wright and Deonarain, 2007; Nelson, 2010).

### Single-chain variable fragment (scFv)

ScFv has high affinity, highly solubility, multi-domains, and high binding specificity with their target antigen and they are used for antibody engineering, biotechnology, cancer research, and biomedical applications. ScFv have been engineered to improve the effector functions of full-length antibodies, carrying toxins to kill cells, or cytokines to activate the immune system. Furthermore, bispecific antibodies have been constructed to target multiple receptors (AlDeghaither et al., 2015). Recombinant antibody engineering and recombinant DNA technology has facilitated successful expression and cloning of widespread antibody fragments in bacteria (*E. coli*), as well as mammalian (Chinese hamster ovary (CHO), or myeloma cell lines e.g., Sp2/0), yeast (*Pichia pastoris*), plant (*Arabidopsis*), and insect cells (*Drosophila melanogaster*) (Frenzel et al., 2013). These smaller fragments have several advantages over full-length antibodies such as tumor and tissue penetration, blood clearance, short retention times, and reduced immunogenicity. Likewise, they have better fusion in bacteria, and display on a filamentous phage. These fragments permit the production of homogenous proteins for diagnostic and therapeutic purposes as well as structural studies (Frenzel et al., 2013; Wu et al., 2016).

ScFv fusion proteins are constructed by the association of heavy (V_H_) and light chains (V_L_) of immunoglobulins via a short peptide linker. Antigen specific scFv can be easily generated by phage display. These fusion proteins have extensive applications in cancer therapeutics such as in lymphatic invasion vessels, colon cancer, hepatocarcinoma, and diagnostics of human disease (Tonelli et al., 2012). Moreover, scFv have been widely used with phage display panning i.e., an affinity selection technique, to construct ligands to detect toxins produced by various pathogenic entities *in vitro* or *in vivo*. Similarly, co-expression vector systems have been used to prevent and cure diseases by scFv. Currently, various scFv fragments have been constructed against toxins and virulence factors of pathogens (Tonelli et al., 2012; Wang et al., 2013, 2016). Additionally, a method for rapid and effective high-affinity GFP-based antibody production corresponding to scFv was developed. It was demonstrated by inserting CDR3 into green fluorescence protein (GFP) loops for improved disease diagnosis and therapy (Wang et al., 2014b). Skp co-expressing scFv has high solubility and binding activity to antigen thermolabile hemolysin (TLH) (a pathogenic factor of *Vibrio parahaemolyticus)* and was developed by using pACYC-Duet-skp co-expression vector. This scFv was constructed to detect TLH directly in real samples. Furthermore, an antibody was developed against TLH that showed strong neutralizing effects on TLH-induced cell toxicity (Wang et al., 2006, 2007, 2012, 2013, 2014a; Chen et al., 2015).

#### Library construction of scFv

Phage-displayed antibody libraries have been widely used for the construction of high-affinity target-specific antibodies (Chen et al., 2008). ScFv is a non-covalent heterodimer that consists of V_H_ and V_L_ domains. Moreover, antibody repertoires from phage-displayed libraries are constructed by harvesting messenger ribonucleic acids (mRNAs) from peripheral blood lymphocytes, hybridoma, spleen, bone marrow, tonsil, and similar other sources (Chen et al., 2008). Large libraries with a diverse range of antibodies and genes are created using reverse transcribed (RT) process into cDNA to function as a template for antibody gene amplification (PCR) (Lim et al., 2010).

Libraries are also created by PCR assembly, phagemid, and sequential cloning or combinatorial infection. The V_H_ and V_L_ chains are combined (linker orientation dependent) and cloned to construct a combinatorial scFv library for antigen selection (Ahmad et al., 2012). Another technique for recombinant antibody production is the utilization of phage recombinants displaying antibodies at their tips, and which undergo biopanning for the *in vitro* selection of scFv from large libraries of variable domains circumventing the traditional hybridoma method. Numerous scFv fragments have been constructed against haptens (Wang et al., 2006, 2014a), proteins, carbohydrates, receptors, and tumor antigens for medical therapies and diagnostic applications (Wang et al., 2012, 2013).

#### Screening by phage display

Phage display is a powerful biological technique for screening specific peptides or proteins. Screening of antibody libraries by phage display permits the rapid selection of scFvs to isolate V_H_ and V_L_ chains for mAb transformation. Thus, therapeutic antibodies against noxious or highly conserved antigens, plasma membrane proteins, and receptors can be obtained in their native conformation while avoiding animal immunization (Rader and Barbas, 1997). The peptide libraries are incubated on a plate coated with the antigen of interest. Next, unbound phages are washed away. Then, the phages are amplified using host bacteria for high-affinity elution and binding/amplification cycles to expand the pool in favor of binding sequences. Finally, individual clones are characterized after 3–5 rounds by DNA sequencing and ELISA to achieve the resultant structural and functional details (Catanese et al., 2007).

#### Expression of scFv

Numerous expression systems such as *E. coli*, yeast, mammalian, insect cell, wheat germ cell-free expression system, and plant-based expression system have been used for the successful isolation of scFv and display as fragments (Wang et al., 2013). The expression and activation of scFv is performed by appropriate folding and *in vitro* refolding for aggregation. The expression systems for the production of active scFv antibody are selected, designed, and constructed based on hosts. The bacterial expression system is the most suitable and widely used method for the production of scFv antibody fragments compared to other available expression strategies (Frenzel et al., 2013).

*E. coli* is a valuable tool for expression systems in the fields of genetics and biochemistry. The system has numerous advantages including enhanced folding, low cost, high throughput, well-studied physiology and genetics, rapid growth, high yields up to 10–30% of total cellular protein, and simple handling. Additionally, it can be used for multi-plexed cloning, expression, and purification of proteins for structural genomics (Rosano and Ceccarelli, 2014).

#### Characterization of scFv

Antibody characterization involves peptide mapping, glycan characterization analysis, purification, fragmentation of antibody pharmacokinetics, and quality assurance for many applications in basic research (Roth et al., 2012). The scFv antibody is usually characterized by affinity, isotype, cross binding, phage-ELISA, and immunoblot. Biochemical characterization includes the expression of scFv antibody in a soluble form in infected *E. coli* cells. In addition, further characterization is carried out by immunofluorescence antibody test (IFAT), mass spectrometry, sequencing and indirect immunofluorescence (IFI) assays, cytotoxicity analysis, surface plasmon resonance, and NMR spectroscopy (Wang et al., 2012; Yuasa et al., 2014; Levenhagen et al., 2015).

### Other antibody fragments

Antibody engineering has become an aesthete discipline covering a wide range of production technologies. Moreover, techniques include the modification of clinically significant therapeutic drugs by antibody fragments especially for clinical imaging and to target multiple disease associated antigens (Spiess et al., 2015).

#### Fab

Fab antibody fragments are smaller with better tissue and tumor penetration than intact mAbs. Fab lack a constant region and therefore, antibody effector functions (Nelson, 2010). Moreover, Fab bind to specific antigens and are used in non-clinical studies (e.g., staining experiments) and clinical therapeutics such as anti-TNFα PEGylated fab fragment. This fragment has a 14 day serum half-life and is used to improve anti-tumor activity and to reduce immunogenicity (Chames et al., 2009).

#### Bispecific antibody

The tumor necrosis factor (TNF) and interleukins 1 and 6 (IL-1 and IL-6) proinflammatory cytokines cause multifactorial disease such as cancer and systemic inflammations. Moreover, these factors are involved in redundancy of disease-mediation and crosstalk between signal cascades (Arango Duque and Descoteaux, 2014). Similarly, upregulation of alternative receptors and pathway switching is often related to resistance to therapy (Dong et al., 2011). The obstruction of several targets or multiple sites on one target is associated with improved therapeutic efficacy. Over the past decade, dual targeting with bispecific antibodies has gradually switched to combinatorial or cocktail therapy. This targeting technique is based on the targeting of multiple disease-modifying reagents with one drug. Several bispecific molecules such as diabodies, IgG-like tetravalent Di-diabodies, IgG-scFv fusion proteins, and bispecific Adnectins™ have been developed to target tumor mediated receptors such as members of the epidermal growth factor (EGF) receptor family, i.e., EGFR, HER2, HER3, and HER4,45 and the insulin-like growth factor 1 receptor (IGF-1R; Tao et al., 2007; Kontermann and Brinkmann, 2015). The application of a single, bispecific molecule is advantageous because it is less complicated to administer to patients, requires reduced preclinical and clinical testing, and has cost effective manufacturing (Kontermann and Brinkmann, 2015).

### Other gene engineered antibody fragments

Smaller antibody fragments permit in depth tissue penetration associated with the affinity of the antibody fragments. Moreover, a high concentration of complete antibody restricts its ability to infiltrate tumors (Weiner and Adams, 2000). Additional engineered antibody fragments include CDRs, Fab, F(ab')_2_, monospecific Fab_2_, bispecific Fab_2_, trispecific Fab_3_, monovalent IgG, bispecific diabody, trispecific triabody, scFv-Fc, minibody, new antigen receptor (IgNAR), variable new antigen receptor (V-NAR) domains in sharks, camelid heavy chain IgG (hcIgG), and VhH (Nelson, 2010; Rodrigo et al., 2015).

## Phage display systems

**Figure 4:**
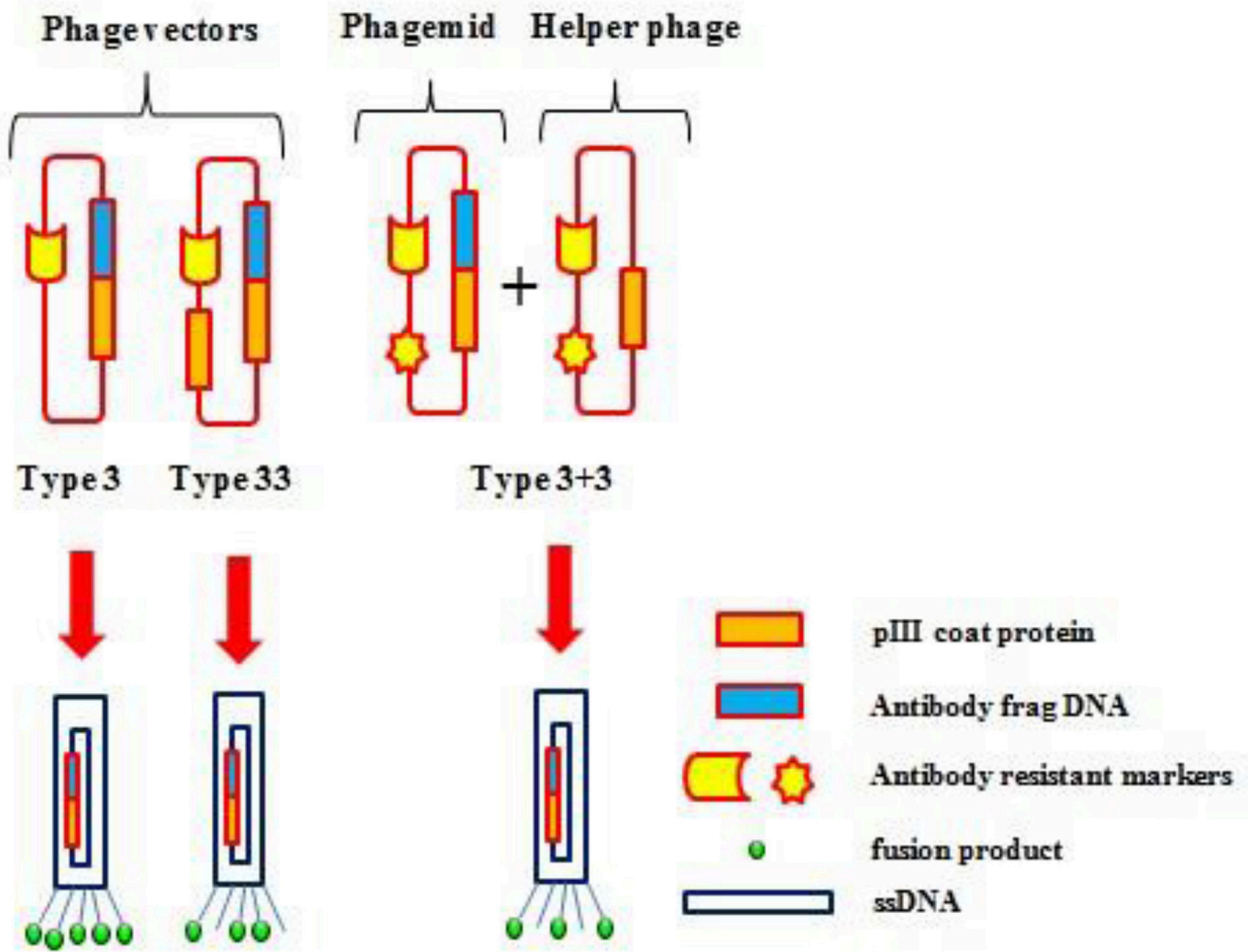
**Various phage display systems**. Gene pIII is represented as an orange box, the foreign DNA insert as a blue box, and the fusion products as a green circle.

Phage display is a selection technique for fusion proteins and phage coat proteins that are expressed on the phage surface. The library is developed by careful genetic manipulation (Figure 4; Chan et al., 2014). Peptide or protein coding genes are inserted into a vector fused to the 5′-terminal of pIII or pVIII that are phage coat proteins. The bacteria are transformed with phagemid libraries, and then infected with a helper phage to assemble phage particles that express fusion proteins on their surface. Subsequently, the displayed proteins/antibody fragments are rooted to the surface of the coat protein, and permit affinity purification with its analogous genes (Barbas et al., 1991; Chan et al., 2014).

### Principles of phage display

Filamentous bacteriophages used in phage display techniques are viruses that belong to the *Inoviridae* family. There are fewer of these filamentous phages in this genus compared with tailed phages. *Inovirus* virions are 7 mm in diameter, contain circular DNA enclosed in a protein capsid, and infect both Gram negative and positive bacteria. They do not lyse host cells, instead, they are packed and extrude at the surface (Marvin et al., 2014).

The genomes of these viruses consist of double strand DNA (dsDNA), single stranded DNA (ssDNA), double strand RNA (dsRNA), and single strand RNA (ssRNA). The viruses enter host cells via pilli and are involved in genome replication, and virion structure, assembly and regulation (Stassen et al., 1994). These viruses undergo extensive recombination, act as vectors (phagemid) for gene transfer and are closely related phenotypically and genotypically. They can integrate into the host genome by phage-encoded transposases and host-encoded XerC/D (Hassan et al., 2010). Filamentous phages have three distinct classes, including M13, f1, fd, and M13 that infects *E. coli*. Most proteins are displayed at phage proteins pIII (fusion) and pVIII (preserving functional coupling with 6–7 residues; Hess et al., 2012).

This phage can be easily manipulated due to its small genome. Therefore, these are the best-studied viruses and are extensively used in phage display technology. Large phage particles can be produced by the insertion of DNA into non-essential regions, are stable in extreme conditions, and can be produced in high amounts (Jończyk et al., 2011).

### Phage display technology (PDT)

A number of *in vitro* techniques have been established for the development of antibodies in comparison with *in vivo* methods that involve animal immunization. PDT is a powerful tool for screening of specific recombinant protein binders against a large number of target antigens, including peptides, glycoproteins, glycolipids, saccharides, nucleic acids, and haptens. PDT is the most commonly used *in vitro* technology (Bradbury et al., 2003a). A feature of this technology is a direct physical link between genotype and phenotype of the displayed protein/variable antibody fragments. Screening of the displayed protein by antigen *in vitro* is analogous to the selection of protein fragments in natural immunity (Petrenko and Vodyanoy, 2003).

#### Production method

**Figure 5:**
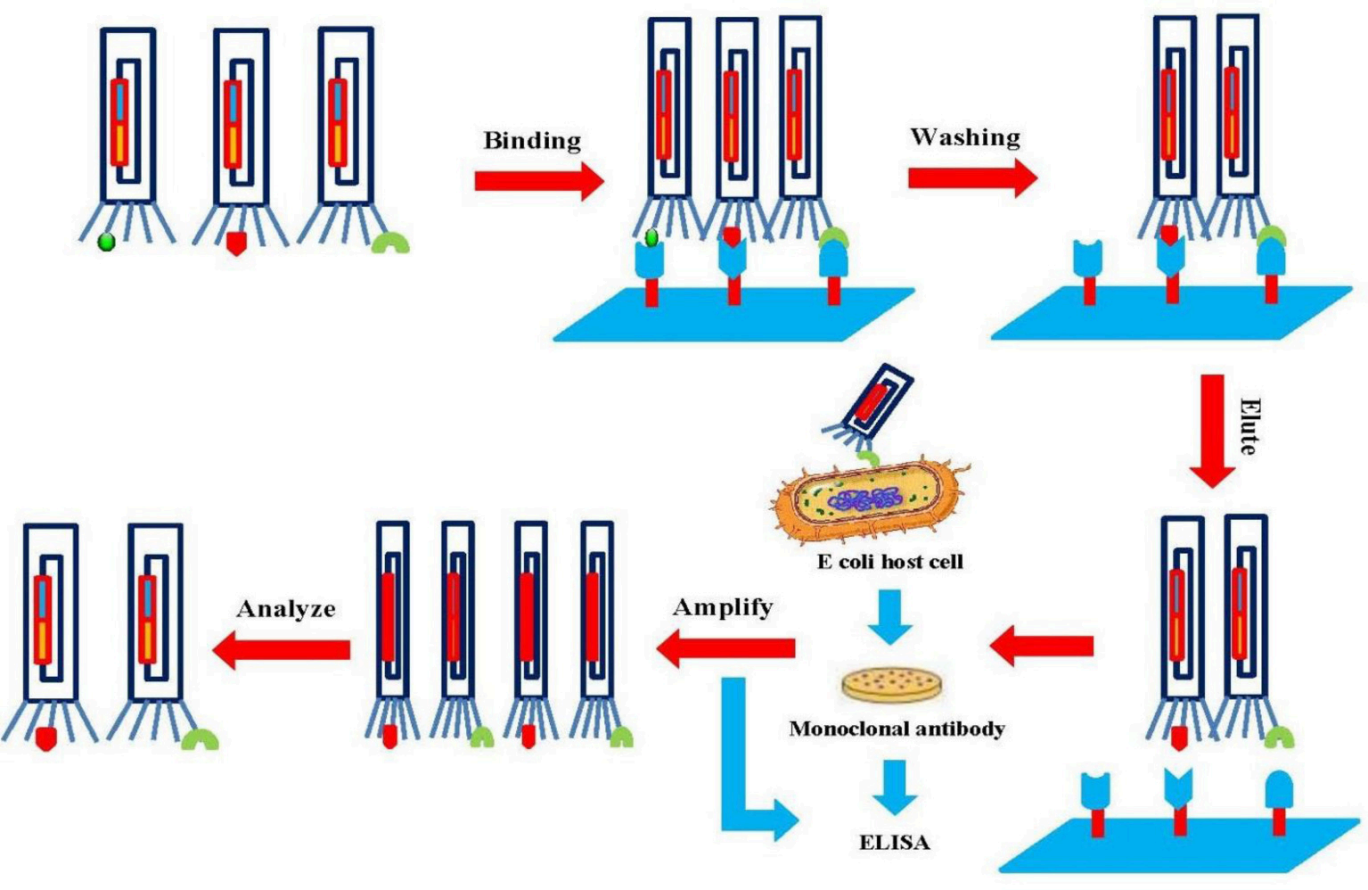
**Schematic illustration of the biopanning technique**. The target is attached to a phage library that is immobilized on a solid surface. Unbound phages are washed out, and specific phages are eluted and amplified. After several rounds of biopanning, the phages are analyzed to obtain diagnostic and therapeutic agents.

Phage display technology has facilitated the production of protein libraries, which are formed with large numbers of phage particles displaying different molecules (10^6^–10^11^ different ligands in a population of >10^12^ phage molecules). Specific binder screening with biopanning allows the enrichment of the desired molecule (Bazan et al., 2012). The first step is the incubation of the display library with an immobilized surface (for example, microplate, magnetic beads, column matrix, PVDF membrane, or immunotubes) of the entire cell. The non-binding phages are then removed by extensive washing and the binders are eluted by acid or salt buffer. Then, binders are amplified using an appropriate bacterial host cell such as *E. coli*. To obtain high-affinity targets, up to five rounds of biopanning are performed (Figure 5). Finally, DNA sequencing of the primary structure is carried out to produce individual clones of the target protein (Bazan et al., 2012; Wu et al., 2016).

Many factors including proper biopanning design, type of immobilized surface, binding time, washing, and antigen concentration affect the level of selection and the screening of antibodies to unique epitopes (Bazan et al., 2012). Numerous screening cycles are essential in biopanning to attain the preferred binding activity of the acquired monoclonal phage antibodies. Several tests including ELISA, fluorometric microvolume assay technology (FMAT), and chromophore-assisted laser inactivation (CALI) are used to analyze this activity (Tonelli et al., 2012).

Various types of phage libraries are used for the screening of specific recombinant proteins/antibodies in relation to antigen specificity: (1) construction of specific high-affinity antibody library for specific antigens by the use of immunized animals with a dissociation constant in the nanomolar range, (2) a single-pot (general) non-specific library produced against antigens, and (3) construction of secondary mutant antibody phage libraries for the screening of antibodies with high specificity (Hust and Dubel, 2004). The development of an antibody library from immunized animals has become obsolete because of the construction of diverse universal single-pot libraries for the isolation of numerous antigens. PDT has many applications in biotechnology, production of recombinant multifunctional antibodies, cancer, immunotherapeutics, and the enhanced validity of protein fragments (Yau et al., 2003).

### Applications of PDT

The study of epitopes and mimotopes in the interactions of antigen-antibody binding was the earliest application of PDT (Wu et al., 2016). Mimotopes are miniscule peptides that mimic linear, intermittent, or non-peptide epitopes. It was noted that scFv, Fab fragment, and VHH domains could be displayed on the phage successfully (Tonelli et al., 2012). This laid the foundation of new molecular recognition techniques to determine protein folding, stability, structure-to-function relationships, and other related protein-protein interactions. The fusion of many ligands with phage particles has enrich phage displayed cDNA libraries significantly (Vithayathil et al., 2011).

Several novel molecular techniques have been established for screening functional molecules. These techniques include the identification of peptide agonists, receptor antagonists, the determination of binding specificity of domains, mapping of simple carbohydrates and functional epitopes, the identification of tumor inhibitor targets, and molecular imaging by fluorescently labeled phages (Fukuda, 2012). Recently, PDT has been widely used in medical sciences for the production of a large number of humanized antibodies and the production of new therapeutics. These antibodies have preclinical and clinical applications (Rothe et al., 2006).

#### Transfusion medicine

A large number of antibody reagents are being developed for hematological applications such as cell subpopulation identification, directed therapeutics, and *in vivo* imaging. Anti-ABO, anti-Rh, and anti-Kell hemagglutination antibodies have been developed against red blood antigens (Marks et al., 1993). Anti-Rh (D) and anti-HPA-Ia bispecific diabodies developed by PDT that are useful for hemagglutination assays. These diabodies are being used for the treatment of neonatal alloimmune thrombocytopenia (Watkins et al., 1999).

Moreover, various antibody reagents have been raised against fetal red blood cells (Huie et al., 2001). Additionally, this technique has helped the production of antibodies against dendritic cells, white blood cells (WBC) (Fitting et al., 2011), hairy cell leukemia (Kreitman et al., 2012), myeloma protein (paraproteins) (O'Nuallain et al., 2007), B and T cells (Maeda et al., 2009), clotting factors, AITP, GPIa, and GPIIIa antigens, CD antigens (Chu et al., 2006), and 11-dehydro-thromboxane B2 (11D-TX) antigens (Siegel et al., 2003).

#### Autoimmune diseases and neurological therapeutics

Human immune libraries developed by PDT facilitate the study of autoimmune and neurological disorder physiology, clinical diagnostics, and the treatment of AITP (platelet disorder caused by anti-platelet autoantibodies), MS, myasthenia gravis (MG) [antibodies against nicotinic acetylcholine receptor (AcChoR)], thrombotic thrombocytopenic purpura (TTP), Cogan's syndrome (CS) caused by systemic vasculitis, acute anterior uveitis (AAU), ocular inflammation, insulin dependent diabetes mellitus (IDDM) caused by the destruction of pancreatic beta cells, Wegener's granulomatosis (Finnern et al., 1997), autoimmune thyroid disease (Latrofa et al., 2003), primary biliary cirrhosis (PBC), and Sjögren's syndrome (SS). Additionally, the technique has therapeutic uses in blistering skin diseases, pemphigus vulgaris (PV) (Payne et al., 2005), pemphigus foliaceus (PF) (Ishii et al., 2008), autoimmune hepatitis (AIH), primary biliary cirrhosis (PBC), mixed cryoglobulinemia (CryoII), systemic sclerosis (SSc), autoimmune cholangitis (AIC), antiphospholipid syndrome (APS), vitiligo rheumatoid arthritis, Crohn's disease, Graves' disease (GD), and celiac disease (genetic inflammatory disorder) (Zohreh and Hossein, 2008).

Neurological disorders are treated by intracellular antibody fragments (intrabodies), which are potentially therapeutic. Intrabodies select abnormal intracellular proteins. However, there are several limitations in the extracellular binding and internalization of DNA transfected by viral based vectors, lipofection or electroporation (Jazi et al., 2012). These are not efficient *in vivo* techniques and can alter cell viability. This problem can be overcome by fusing protein transduction domains (PTD) to antibodies (Langedijk et al., 2004). Phage display libraries have been utilized for novel immunotherapeutic strategies for the treatment of neurotoxins, Creutzfeldt–Jakob disease (CJD), and Gerstmann-Sträussler-Scheinker syndrome (GSS). They are also used for kuru disease, familial fatal insomnia by the accumulation of abnormal prion protein (PrPSc) (Thanongsaksrikul and Chaicumpa, 2011), Huntington's disease, and Parkinson's disease. Moreover, they have been employed in inhibitory studies of β-amyloid formation, and enzyme therapy of the brain vasculature and brain parenchyma (Chen et al., 2009).

#### Peptide homing in organs and molecular imaging

Biopanning *in vivo* with phage display libraries has facilitated the isolation of peptides homing to all types of organs in the human body. Phage display is applied to stem cells for cell based regenerative medicine (Gothard et al., 2011). Moreover, this technique has assisted in the guided delivery of various peptides/drugs such as proapoptotic peptides, cytotoxic drugs, metalloprotease inhibitors, and cytokines to specific targets (Nixon et al., 2014). The binding of peptides with the extracellular domain of the LOX-1 receptor is associated with hypertension and atherogenesis (Nixon et al., 2014). Other studies have reported that the homing of an RGD-motif-containing peptide to angiogenic vasculature was linked to a proapoptotic peptide and was successfully used for the treatment of collagen-induced arthritis in mice. Phage libraries have also been used for anti-obesity, microparticle (MP), avb3 integrin angiogenesis therapy, and in targeting vascular endothelial growth factor (VEGF) (Cooke et al., 2001).

Similarly, phage displays are used for tumor targeting agents e.g., the scFv (MFE-23) molecule is specific for carcino embryonic antigen (CEA) (Edwards et al., 2008). This technique has replaced radiolabeled antibodies that have multiple disadvantages including reduced natural immunity (Adachi et al., 2011). Furthermore, PDT has been used to isolate a number of peptides for molecular imaging. Its advantages are small size, rapid blood clearance, lack of immunogenicity, tissue penetration, and increased diffusion. Numerous peptides for tumor targeting were isolated using human B cell lymphoma (McGuire et al., 2006), cervical (Robinson et al., 2005), colon (Rasmussen et al., 2002), gastric (Liang et al., 2006), breast (Askoxylakis et al., 2005), lung (Chang et al., 2009), glioblastoma (Wu et al., 2008), hepatic (Du et al., 2006), prostate (Zitzmann et al., 2005), neuroblastoma (Askoxylakis et al., 2006), and thyroid (Zitzmann et al., 2007) carcinoma cell cultures. However, about 80% of these peptides have not been reported to function *in vivo*. This inactivity was observed in peptides that recognized mouse double minute 2 homolog-p53 protein (MDM2/p53) (Pazgier et al., 2009), IL-11 receptor (Zurita et al., 2004), prostate specific antigen (PSA) (Pakkala et al., 2004), heat shock protein 90 (Kim et al., 2006), and growth factors (Hetian et al., 2002).

## Hybridoma technology vs. PDT

Hybridoma technology is a well-established method for the generation of murine mAb cell lines by the fusion of splenocytes (harvested from immunized mice) with myeloma cells. The technology remains a feasible method for laboratories that implement basic cell biological research. Hybridoma technology is a comparatively simple procedure with minimal cost for the steady production of native whole immunoglobulins (Tomita and Tsumoto, 2011). Nevertheless, this technology has various limitations such as antibodies produced by the hybridoma technique are strictly murine proteins that limits their therapeutic use in humans. In addition, they also trigger human anti-mouse antibody (HAMA) responses (Tjandra et al., 1990). Moreover, indefinite production costs, low fusion efficiency, limited number of mAbs, difficulty in developing mAbs against strictly conserved and toxin antigens and time consumption are other disadvantages (Hnasko and Stanker, 2015).

The production and amplification of antibodies *in vitro* using bacteria by PDT has a low turnaround time compared with other methods. Additionally, the library comprises of diverse variants up to 10^13^, which can be selected against a varied range of biological and inorganic targets (Sblattero and Bradbury, 2000). The experimental conditions can be controlled, and the required equipment and libraries are available commercially. Disadvantages include a difficult procedure and lack of antibodies displayed on the surface of each bacteriophage yielding a small number of mAbs (Willats, 2002).

## Other antibody engineering techniques

Antibody engineering is a remarkable modification technique for production of highly specific and efficient antibody products. However, antibodies are bulky macromolecules that encompass challenges in construction, optimal pharmacokinetics, manufacturing, stability, and process development. Nonetheless, progress in antibody engineering technologies such as phage display, yeast display, bacterial display, and ribosomal or cell-free display continue to advance our capacity to rapidly screen and refine stable binding immunoglobins. These engineering techniques further improve biological properties significantly in the effector domains of the mAbs (Filpula, 2007).

### Engineered immunomodulatory antibodies

Immunomodulatory techniques are persistently progressing to expand the clinical efficacy of therapeutic antibodies. Cell surface antigens exhibit a wide array of targets that are overexpressed, mutated or selectively expressed, and selected for modulated antibody-based therapeutics. The technology functions through engineering alterations in antigen or receptor function, the immune system i.e., altering Fc function and T cell activation and antibody conjugated drug delivery system (DDS) targeting a specific antigen. Immunomodulatory antibodies have gained significant clinical success (Scott et al., 2012).

The Fc region is modulated by engineering the effector function, for example to increase or lessen binding to Fc gamma receptors (FcγRs) or complement factors and the half-life of IgG. The half-life can be extended by improving affinity of Fc for Fc neonatal receptor (FcRn). Moreover, it can be prolonged by engineering pH-dependent antigen binding to enhance recycling of IgG via FcRn, and effective binding to the target molecule. Engineering the Fc region permits the development of molecules that are better suited to the pharmacology activity required of them (Vincent and Zurini, 2012; Rath et al., 2015). Recently, a study investigates engineering the pH-dependent interaction between IgG and FcRn. It involves modulation of constant Fc part of monoclonal human IgG1 (hIgG1) antibodies to improve effector functions and clinical efficacy of next-generation IgG1-based therapeutics (Grevys et al., 2015).

**Figure 6:**
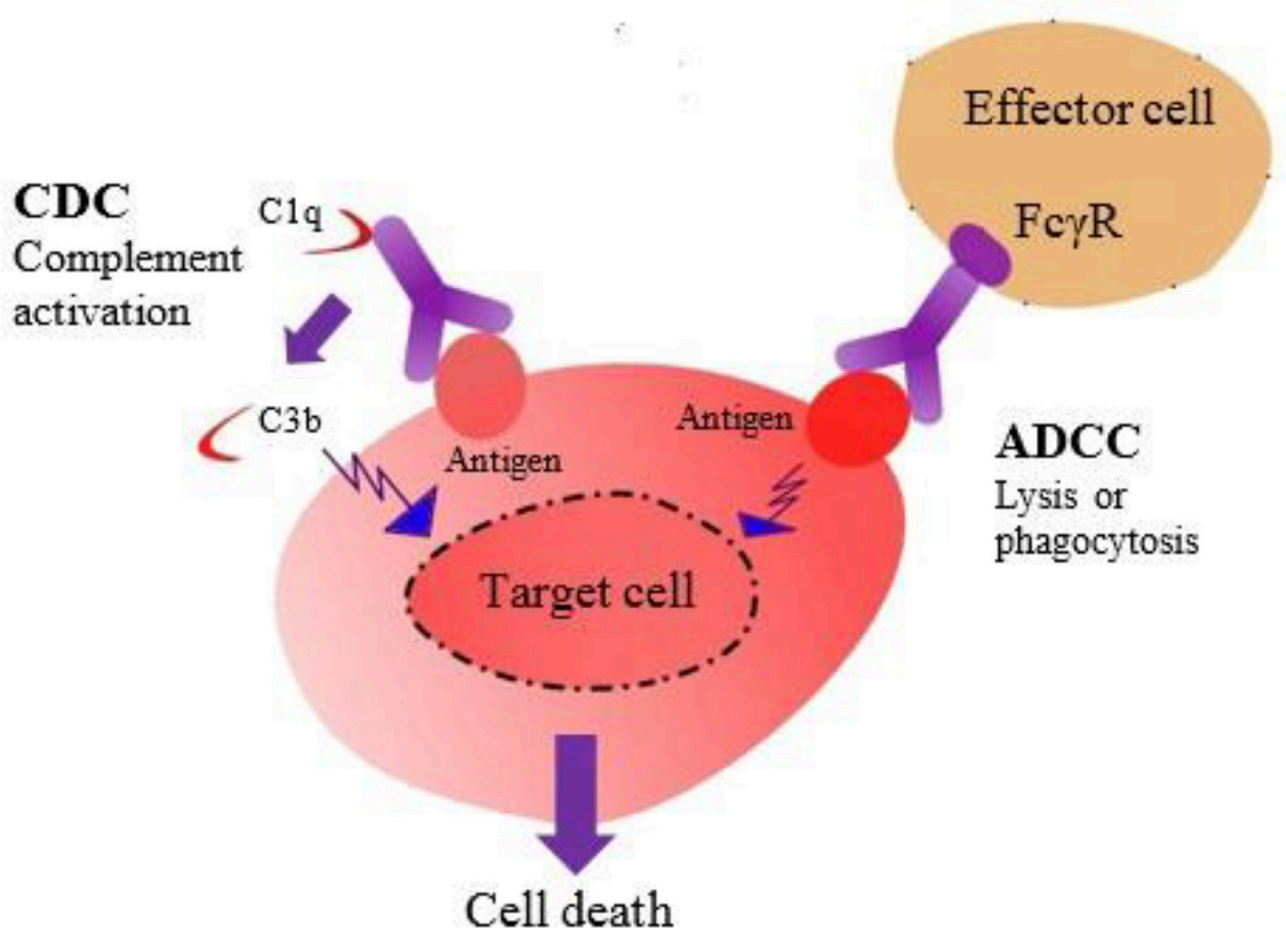
**The Fc region of an antibody mediates effector functions such as CDC and ADCC**.

Similarly, new opportunities have been created by the development of antibody-drug conjugates (ADCs) to treat the infectious diseases or target cancer cells. ADCs are being developed by progressing in antibody generation, selection of exceedingly cytotoxic molecules, and construction of stable linkers that can be investigated in clinical trials (Vincent and Zurini, 2012). Cytotoxic therapeutic mAbs often help target cell-killing by eliciting immune effector functions. These include antibody-dependent cell-mediated cytotoxicity (ADCC), antibody-dependent cellular phagocytosis (ADCP) mediated by innate immune effector cells, and complement-dependent cytotoxicity (CDC) mediated by humoral components (Figure 6). *in vitro* studies, Fc engineering methods have been specifically designed to modulate ADCC, ADCP, and CDC envisioned for therapeutic mediation (Kinder et al., 2015).

Natural killer (NK) cells exhibit essential role in immunity in the context of mAb treatment by exerting direct cytotoxicity toward infected or tumor cells and contributing in modeling the adaptive response (Cheng et al., 2013). Several T- or NK-cell modulators such as ipilimumab and nivolumab were approved for the treatment of metastatic melanoma (Berman et al., 2015).

Fc-engineered antibodies improve the ADCC/ADCP potential and target CD19, CD20, CD40, and Her2. Consequently, they enhance the therapeutic potential of mAbs. NK cells are exclusive in exhibiting low-affinity activating FcγRIIIa (CD16), and no inhibitory antibody receptors, featuring a substantial role in ADCC. Several studies have established a link between activating Fc receptors and the efficacy of mAb therapy using mouse tumor models (Romain et al., 2014).

Recently, glyco-engineering technique has been used to produce recombinant therapeutic proteins with optimized efficacy, half-life, specificity, and antigenicity. Glyco-engineering of expression platforms is progressively documented as an essential approach to advance biopharmaceuticals (Ferrer-Miralles et al., 2009; Dicker and Strasser, 2015). The technique has been applied to *in vivo* expression systems that include mammalian cells, insect cells, yeast, and plants for the production of recombinant proteins. The underlined approaches aim at developing glycoproteins with homogeneous N- and O-linked glycans of defined composition (Dicker and Strasser, 2015). Moreover, multi-level glyco-engineering techniques have been investigated to generate IgG with defined Fc-glycans in eukaryotic cells (Dekkers et al., 2016). Additionally, *E. coli* expression have been successfully employed to produce recombinant human interleukin-2 (IL-2) (Kamionka, 2011).

### Yeast, bacterial, and ribosomal display techniques

Antibodies are engineered with superior properties such as binding affinity, stability, and catalytic activity by several other display tools (for example, yeast and bacterial display) for broad spectrum of biotechnology, medicine, and biomedical applications. Yeast surface display exhibit development of recombinant antibodies by displaying on the surface of *Saccharomyces cerevisiae* via genetic fusion to an abundant cell wall protein (Cherf and Cochran, 2015).

Yeast display technique has been used for engineering protein affinity, stability, and enzymatic activity. Moreover, it is extensively applied in protein epitope mapping, identification of protein-protein interactions, and uses of displayed proteins in industry and medicine (Cherf and Cochran, 2015). Several recombinant antibodies have been generated by yeast display for lethal infections such as highly pathogenic H5N1 avian influenza virus (Lei et al., 2016), cell tumor (Li et al., 2017) and human tumor endothelial marker 1 (TEM1) (Yuan et al., 2017).

Similarly, several bacterial display systems have been established for Gram-negative bacteria and Gram-positive bacteria (Lee et al., 2003). The display systems comprised of a carrier protein as an anchoring motif, a target protein, and a host strain. Proteins developed for use as anchoring motifs include outer membrane proteins, lipoproteins, autotransporters, subunits of surface appendages, and S-layer proteins (Han and Lee, 2015). Bacterial display has widespread applications including live vaccine development, screening-displayed peptide libraries, biosorbents, whole-cell biocatalysts, and biosensor development. Moreover, the promising technology is helping in the remediation of pollutants, biofuel production, and production of enantiomerically pure compounds (Han and Lee, 2015; Ramanan et al., 2016).

Ribosome display is a cell-free display system, and a technique to perform entirely *in vitro* selection of proteins or peptides to bind desired ligand. Ribosome display consists of both prokaryotic and eukaryotic display systems (Zahnd et al., 2007). It forms stable protein-ribosome-mRNA (PRM) complexes and links individual nascent proteins (phenotypes) to their analogous messenger RNA (mRNA) (genotypes). Ribosome display allows synchronized isolation of a functional nascent protein, through affinity for a ligand together with the encoding mRNA. The encoding mRNA is then transformed and amplified as DNA for further manipulation, including repeated cycles or protein expression. The advantages of ribosome display over other cell based methods include displaying very large libraries, generating toxic, proteolytically sensitive and unstable proteins, and incorporation of modified amino acids or mutations at distinct positions (He and Taussig, 2002; Zahnd et al., 2007). Ribosome display systems have been investigated to identify potential antigens of *Clonorchis sinensis* (Kasi et al., 2017), and human tumor necrosis factor α (hTNFα) for diagnosis and treatment (Zhao et al., 2016).

### Advantages and disadvantages

The large size of mAbs limits tumor penetration, and their long serum half-life is not suitable for therapy and imaging applications. Therefore, antibody fragments have been constructed in various formats as they are small, monovalent, penetrate tumor tissues efficiently, and are rapidly eliminated by renal clearance (Chames et al., 2009).

Similarly, recombinant antibodies have several advantages: (i) bacteria, yeast, plants, or animals can be used to produce antibodies, (ii) no need for immunization, and (iii) intrinsic properties (immunogenicity, binding affinity, pharmacokinetics, specificity, and stability of antibodies) can be modified easily using mutagenesis techniques. Genetically engineered antibodies have integral characteristics that suit various downstream applications or can be converted into functional whole immunoglobulins (Bradbury et al., 2003b). Antibodies exhibit strong immunity to defend against foreign antigens and non-self-agents. However, a variety of recombinant antibodies is needed to interact these hostile antigens. Over the last decade, the use of antibody engineering or recombinant antibody technology has shaped the genetic manipulation of a diverse range of antibody fragments for research, diagnosis, and therapy (Kontermann and Muller, 1999). This technology has resulted in better affinity and specificity of manipulated antibody fragments and has facilitated the replacement of hybridoma technology with various display systems for unlimited antibody production against any known antigen (Gram et al., 1992).

Conversely, engineered antibodies have various disadvantages such as they exhibit greater expense and complexity in manufacturing compared to antibodies developed by hybridoma technology (Spiess et al., 2015). Due to their foreign nature, engineered therapeutic antibodies lead to allotypic immune responses that results in rapid clearance from body by kidney, elicit on T-cell help, and have reduced antibody avidity. Moreover, engineered antibodies exhibit reduced half-life due to lack of an Fc domain and prevention of FcRn-mediated recycling. Likewise, antibody based therapies have more limitations based on the fact that many targets (sometimes in low level) have not yet been determined for various diseases (Chames et al., 2009; Attarwala, 2010).

## Applications of antibodies

In the 1890s, von Behring and Kitasato worked on tetanus antitoxin that lead to the development of a new discipline, immunology. They described antibodies for the first time and discovered that inactive toxins can elicit a protective immune response against active toxins in animals (Kantha, 1991). The transfusion of serum from these protected animals elicited an immune response in other animals. Therefore, antibodies were originally called “antitoxins.” Since then, antibodies have been shown to have a wider repertoire of antigen recognition. Antibodies are widely used in diagnostic tests referred as “immunoassays.” These are used to confirm diagnoses and for rapidly growing antibody based technologies (Mukherjee et al., 2012).

Similarly, antibodies have remarkable applications in the field of diagnostics, therapeutics and targeted DDS. They have been used to study various diseases such as cancer, metabolic and hormonal disorders, and infections caused by bacteria, viruses, fungi, algae, protozoa and other agents (Sundar and Prajapati, 2012). Moreover, these biomolecules have numerous application in the diagnosis of lymphoid and myeloid malignancies. Likewise, immunoglobins are used in tissue typing, ELISA, radio immunoassay, serotyping of microorganisms, immunological intervention with passive antibody, analyzing a patient's antibody profile, and antiidiotype inhibition (Siddiqui, 2010).

### Detection and immunodiagnostic test kits

The detection of antibodies against infectious agents and the construction of rapid and sensitive antibody-based immunodiagnostic test kits is an important part of basic and clinical research. Assays have been developed against pathogens, toxins, and infectious proteins/peptides (Ahmad et al., 2012). The production of antibodies depends on specific protein titers, extent of immunity, and identification of B cell responses (Burbelo et al., 2010). Antibodies are routinely detected by ELISA and other immunoassays. Accessibility of full length DNA sequences is useful for the rapid and robust systematic identification of antigens by recombinant proteins using protein arrays for complete proteome analysis (Kierny et al., 2012). Furthermore, numerous novel high-throughput dominant immunoassays are used to detect antibody responses against antigens (Burbelo et al., 2010).

#### Enzyme-linked immunosorbent assay (ELISA)

ELISA is the most common method for the quantitation of pathological antigens. In some cases, modified ELISA have been used in combination with various specific proteins/peptides. It is rapid, consistent, relatively easy to analyze, and adaptable to high-throughput screening (Alonso et al., 2015).

Principally, specific antibody is immobilized on high binding ELISA plates by incubation overnight at 4°C or for 1–2 h at 37°C, and then followed by 3–5 washes with PBST (137 mM NaCl, 2.7 mM KCl, 8.1 mM Na_2_HPO_4_, 1.5 mM KH_2_PO_4_, and 0.5% Tween 20; Murayama et al., 1996). Then, plates are blocked with irrelevant protein e.g., albumin (for example, PBS containing 4–5% skimmed milk), and incubate overnight at 4°C or for 1–2 h at 37°C. After washes, samples and standard dilutions are added to the wells to be captured by bound protein, incubate for 1–2 h at 37°C and wash properly. Next, specific peroxidase-conjugated enzyme labeled detection antibody is added to the wells to enable detection of the captured protein and incubate for 1 h at 37°C. After appropriate wash, colorimetric substrate is added to the wells and incubated for 15–20 min at 37°C for the color development as catalyzed by the enzyme. For instance, addition of 3,3′,5,5′-tetramethylbenzidine (TMB) substrate develops blue color that can be read by microplate plate reader at the wavelength of 562 nm. Additionally, addition of 0.16–2 M sulfuric acid (H_2_SO_4_) as a stop reaction solution develops yellow color that can be read at 405–450 nm correspondingly. In addition, the incubation conditions and reagent formulations should be preferably optimized based on the type of ELISA (Grange et al., 2014; Thiha and Ibrahim, 2015).

There are four ELISA methods. Direct ELISA (dELISA) uses competition between unwanted proteins for plastic binding spots. Antigen is attached to the solid phase followed by an enzyme-labeled antibody. This assay is used to detect various pathogenic antigens (Brasino et al., 2015).

Indirect ELISA (iELISA) uses an antigen attached to a solid phase followed by the addition of unlabeled primary antibody. Instead, a peroxidase enzyme-conjugated secondary antibody is added onto the first antibody. The iELISA is used to detect specific antibodies in sera (Peng et al., 2016).

Competitive ELISA (cELISA) is used for the detection of small molecules lacking multiple epitopes. Specific antibodies to the analyte of interest are immobilized on a micro-titer plate. Then, enzyme-conjugated antigen is incubated with capture antibody and the same antigen in its unconjugated form. This step yields a color following the addition of substrate. The signals produced are directly proportional to the quantity of conjugated enzyme bound and inversely proportional to the quantity of unconjugated antigen present (Dupont-Deshorgue et al., 2015).

Sandwich ELISA (sELISA) is used for larger proteins with multiple epitopes and two antibodies can be used consecutively. The capture antibody is immobilized on a microtiter plate. Then, unknown or known samples are added into the matrix to minimize attachment to the solid phase. Peroxidase-enzyme labeled antibody is then added for coloration, which is directly proportional to the amount of antigen present (Qu et al., 2016).

ELISA has been extensively used in the detection of various pathological antigens from viral, bacterial, fungal, protozoa, algal, and numerous other sources (Thavaselvam and Vijayaraghavan, 2010). An improved ELISA has been used for detecting anti-melanoma differentiation-associated gene 5 (MDA5) antibodies that are expressed in patients with dermatomyositis. Clinical study of this newly developed ELISA exhibited efficient detection of anti-MDA5 antibodies and showed promising potential to assist the routine clinical check of anti-MDA5 antibodies in patients who supposed to have DM (Sato et al., 2016).

#### Western blot

Western blot assay (WBA) is also called immunoblotting. This technique is used for the determination of molecular weight and amount of relevant proteins present in a sample. Proteins in a sample are first separated by electrophoresis and then transferred to a nitrocellulose or polyvinylidene difluoride (PVDF) membrane for the detection of bound primary proteins with antibodies specific to the protein of interest (Ness et al., 2015).

Nonspecific sites in the membrane are then blocked with BSA, non-fat milk powder, or casein. Finally, a labeled secondary antibody is added for detection by chemiluminescence or fluorescence (Lewis et al., 2016). WBA has been used for the confirmation of presence of purified proteins produced against various pathological antigens such as the lethal toxin of *Clostridium sordellii* (Arya et al., 2013), shiga toxin Stx2f (Skinner et al., 2013), *Staphylococcus aureus* alpha-hemolysin (alpha-toxin) (Ladhani et al., 2001), *Selenocosmia huwena* huwentoxin-IV (HWTX-IV) (Yu et al., 2014), and *V. parahaemolyticus* thermolabile hemolysin (TLH) (Wang et al., 2015).

#### Dot blot

Dot Blot (DB) assays are used to measure protein concentrations semi-quantitatively. It is slightly different from the WBA. Proteins in the sample are not separated by electrophoresis but are spotted directly on a membrane and hybridized with an antibody probe (Emmerich and Cohen, 2015). This technique is cost effective and uses avidin-biotin technology with diaminobenzidine as a chromogen. It is used for the analysis and quantitation of 14-3-3 protein in cerebrospinal fluid (CSF) samples from cases of Creutzfeldt-Jakob disease (CJD), and for disease control of other neurodegenerative diseases such as Alzheimer's disease (AD) and Parkinson's disease (PD) (Subramanian et al., 2016).

#### Immunohistochemistry

Immunohistochemistry (IHC) is used for the detection and identification of proteins and their localization in tissues. It is essential to retain the morphology of tissues, cells and the availability of antigen sites. Fresh, rapidly frozen tissue sections are preferably used for IHC by chemically fixing tissues in formalin and embedding in wax (Zhu et al., 2015). Additionally, fixing crosslinks amino acids in the tissue that block access to the epitope sites and prevent the action of any protein specific antibodies. The exposure of hidden epitope regions is performed by digestion with an enzyme or by heat treatment, which removes endogenous peroxidase activity and non-specific sites are blocked. A labeled antibody or an unlabeled primary antibody specific to the protein of interest is used, followed by the addition of a secondary labeled antibody specific to the primary antibody (Diorio et al., 2016).

IHC has recently been used for determination and identification of expressed proteins such as lysosome-associated protein transmembrane-4 beta (LAPTM4B) associated with the prognosis of several human malignancies (Xiao et al., 2017), glucagon-like peptide-1 (GLP-1) against renal tubular injury (Guo et al., 2017), and CD147 trans-membrane protein that induces expression and activity of matrix metalloproteinases (MMP) (Dana et al., 2016).

#### Immunoprecipitation

Immunoprecipitation (IP) is used for the study of protein-protein interactions, specific enzyme activity, posttranslational modifications, protein quantification, and determination of molecular weight of protein antigens. This technique includes antibody/antigen purification complexes at conditions that specifically bind antibodies. Rare proteins can be accumulated up to 10,000-fold by IP (Dwane and Kiely, 2011). Luciferase immunoprecipitation systems (LIPS) have been developed for the rapid detection of antibodies against peste des petits ruminants virus (PPRV) (Berguido et al., 2016), varicella-zoster virus (VZV) (Cohen et al., 2014), zinc transporter (ZnT8) autoantibodies (Ustinova et al., 2014), and pancreatic and duodenal homeobox 1 autoantibodies (PAA) (Donelan et al., 2013).

#### Flow cytometry

Flow cytometry (FC) is used to study antibodies on the cell surface and their related physiochemical properties. This technique was developed over 40 years ago (Robinson et al., 2012), and allows the rapid detection of various proteins on cells. Proteins produce fluorescence or scattered light when passed through the machine sensing point to generate quantitative data on a large number of cells. Labeled antibodies are used for the detection of target protein molecules or antigens on the surface of cells (Álvarez-Barrientos et al., 2000). FC has been used for the visualization of pulmonary clearance (Zhou et al., 2015), fluorescence decay lifetimes, control sorting (Houston et al., 2010), quantification of DNA-end resection in mammalian cells (Forment et al., 2012), microsphere-based protease assays, high-throughput screening (Saunders et al., 2010a), and botulinum neurotoxin type A light chain protease inhibitors (Saunders et al., 2010b).

#### Fluorescence activated cell sorting

Fluorescence Activated Cell Sorting (FACS) is used for the detection of specific cells from a mixed population of cells based on their distinctive fluorescence or light scattering characteristics (Yilmaz et al., 2010). The technique allows the isolation of cells by flow cytometry. FACS has been used to isolate high-lipid strains of *Tetraselmis suecica* (Montero et al., 2011), high-lipid mutants of *Nannochloropsis* (Doan and Obbard, 2011), *E. coli* O157, (Ozawa et al., 2016), high-lipid *Chlamydomonas* mutants (Terashima et al., 2015), and *Chlorella* (Manandhar-Shrestha and Hildebrand, 2013). In addition, the technology has been used for quantification of the cellular uptake of cell-penetrating peptides and mRNA (Date et al., 2014).

#### Enzyme linked immunospot

Enzyme linked immunospot (Elispot) assay is used for monitoring cellular immune responses in humans and other animals (Whiteside et al., 2003). It involves a polyvinylidene difluoride (PVDF) assisted microtiter plate pre-coated with antibodies specific to the antigen of interest. A capture antibody binds to the analyte of interest under precise conditions. Then, a biotinylated antibody specific to the analyte of interest is added to detect the original antibody after a wash to remove cell debris. Finally, an enzyme labeled conjugate is added after a second wash to remove unbound antibody and to visualize a colored product. The product is typically a black spot representing a single cell that produces the antigen of interest (Janetzki et al., 2005). This technique was used in the development of a coxsackievirus A16 neutralization test (Hou et al., 2015) and the determination of rotavirus infectivity (Li et al., 2014). Diagnostic applications include diagnosing sensitization to house dust mites (Chang et al., 2016), pulmonary tuberculosis (Pang et al., 2016), pleural tuberculosis (Adilistya et al., 2016), smear-negative tuberculosis (Li et al., 2015), spinal tuberculosis (Yuan et al., 2015), and cytomegalovirus infection (Nesher et al., 2016).

#### Lateral flow test

The lateral flow immunochromatographic test (LFT) is a simple and cost effective device used to detect the presence or absence of a target antigen. It is widely used for medical diagnostics at home (pregnancy test) or in a laboratory testing. The technology involves the transportation of fluids (e.g., urine) through capillary beds, fragments of porous paper, microstructured polymers, or sintered polymers (Hansson et al., 2016).

The technique comprises various components and steps. A sample pad acts as a sponge and absorbs the fluids. Then, fluids migrate to a conjugate pad containing protein conjugates immobilized on the surface of bio-active particles in a salt-sugar matrix that reacts with the antigen. Next, sample fluids dissolve the conjugate salt-sugar matrix and antibody-particles. The fluid mix flows through the porous structure causing the analyte to bind with particles while migrating further through the third capillary bed. Finally, there is a third capture molecule in striped areas that binds to the fluid mix containing analytes and the particle consequently changes color (Yu et al., 2012). LFTs can be used as a competitive or sandwich assay. Latex (blue color), nanometer-sized particles of gold (red color), or fluorescent or magnetic labeled particles are also used (Seydack, 2005). The technique is qualitative, nevertheless, the quantity of analyte in a sample can be measured by the intensity of the test line color. It is usually carried out by optical and non-optical lateral flow readers or biosensors (LFBs) such as complementary metal-oxide semiconductor (CMOS) or a charge-coupled device (CCD) and magnetic immunoassay (MIA) (Gui et al., 2014).

### Diagnosis and cure

Progress in hybridoma technology and the production of highly specific mAbs has revolutionized the therapeutic use of antibodies for the diagnosis and cure of infections, the development of vaccines, antigenic characterization, and genetic manipulation. Antibodies have widespread applications in diagnostics, therapeutics and targeted DDS against potent pathogens, cancer, and physiological disorders (Tiwari et al., 2012).

They are used for the diagnosis of lymphoid and myeloid malignancies, tissue characterization, ELISA, radiolabeled immunoassay, and serotyping of microbes. In addition, they are used in the diagnosis of immunological interpolation with passive antibodies, anti-idiotype suppression, or magic bullet treatments with cytotoxic agents coupled to anti-mouse specific antibodies (Keller and Stiehm, 2000). Similarly, recombinant DNA technology (rDNA) has revolutionized the reconstruction of mAbs by genetic engineering using chimeric antibodies, humanized antibodies, CDR grafted antibodies for therapeutic use (Dimitrov and Marks, 2009; Shen et al., 2014; Fitting et al., 2015).

### Imaging

Molecular imaging provides a sensitive, non-invasive method for the molecular characterization of a cell surface phenotype for disease diagnosis and treatment. It is a rapidly growing multidiscipline that involves molecular biology, immunology, and medicine (Massoud and Gambhir, 2003). The therapeutic applications of antibodies include clinical diagnosis and treatment in hematology, cardiology, immunology, autoimmunity, infectious diseases, and oncology (Neves and Kwok, 2015).

MAbs can be used as molecular imaging probes for investigating cell surfaces *in vivo*. Coupling of cell surface targets with advances in antibody technology have facilitated the production of antibodies optimized for non-invasive imaging (Manning et al., 2008). They have numerous applications such as radioimmunoscintigraphy, antibody-based positron emission tomography (immuno-PET), and single-photon emission computed tomography (SPECT or SPET; Knowles and Wu, 2012).

### Epitope mapping

Phage libraries are useful for mapping antibody epitopes, and display millions of peptides/proteins with unique random sequences. Antibodies select peptides according to the affinity of paratopes (their combining sites) from the libraries (Forsstrom et al., 2014).

#### Immuno-protective epitope mapping

Antibodies protect a host against invading microbes. These natural vaccines neutralize toxins and induce microbicidal effector functions. Antibody-mediated identification of pathogens during infection is essential for revealing immunoprotective responses in the host (Sharon et al., 2014). B cell epitope (variable region of protective antibodies in contact with infectious antigens) mapping is important for the development of effective vaccines in support of sero-diagnostics. This technique identifies protective epitopes for vaccine development and the estimation of conventional vaccines (killed or attenuated pathogens; Malito et al., 2015).

B and T cell epitope conformation is determined by pepscan technique, which has led to the development of antibody therapeutics, vaccine design, and recognition of protective antibody responses (Ahmad et al., 2016). The technique also targets pathogens with novel antimicrobials. Examples include the V-shaped Ab52 glycan epitope in the O-antigen of *Francisella tularensis*, the CR6261 peptidic epitope in influenza virus H1N1, and the PG16 glycopeptidic epitope in the gp120 V1/V2 loop of HIV type 1 (Sharon et al., 2014). Peptides are screened by biopanning with antibodies from the sera of various human diseases, including severe acute respiratory syndrome (SARS), human papillomavirus (HPV), and avian influenza viruses (AIV). Moreover, peptide-based antigens are also used for serological diagnosis and development of vaccines (Wu et al., 2016).

#### Surface plasmon resonance

The surface plasmon resonance (SPR) technique was first used in the year 2000, for the epitope mapping of polysaccharides in *L. pneumophila* and the epitope mapping of various low affinity peptide/protein interactions. SPR is analogous to the working principles of the ELISA technique, but it is label free (Säfsten, 2009). This technique screens molecular interactions in real-time with complex physical principles. Binding of recombinant peptide/protein can be analyzed against natural ligands and mAbs (Säfsten, 2009). SPR was used for the epitope mapping of vitamin B12 (holo-transcobalamin), hemophilia disease, ricin toxin and the manganese transport protein C (MntC) of *Staphylococcus* sp. It also identified Goodpasture auto-immune disease epitopes in combination with PDT (Nguyen et al., 2014).

#### Next generation phage display

Phage display panning methods have failed to yield specific results because of the presence of parasitic phage clones that are often not removed by washing. To overcome these limitations, next generation sequencing (NGS) technology is used to sequence sub-libraries in biopanning experiments (Naqid et al., 2016). Next generation phage display (NGPD) can sequence 3,000 up to a million reads per panning round. This method is more rapid compared with traditional ELISA screening. NGPD has been used to identify target specific ligands by a single panning round using a library or ligand motif (Ravn et al., 2013). This technique has widespread applications for the identification of thousands of ligands and mapping antibodies in response to infectious particles. Clinical targets and antigen-related ligands have been discovered with the help of genetically encoded peptide libraries. Correspondingly, ligands are selected using NGPD by the detection of low abundant copy clones without numerous rounds of selection. Subsequently, NGS has facilitated the quantification of gene expression, genome assembly, and metagenome analysis. Additionally, it is used for PDT with the ion torrent technique (Matochko and Derda, 2015).

### Use of antibody in treatment of infectious diseases for healthier future

Antibodies are critical for immunity against infectious diseases, and extensively used for prevention and treatment of infection caused by bacteria, virus, and other infectious agents to improve public health. Emil von Behring was awarded the first Nobel Prize in Physiology or Medicine in 1901 for his discovery of serum therapy for diphtheria. This led to the term antitoxin and later named as “antikörper” translated “antibody” by Paul Erlich in a 1891 paper (Graham and Ambrosino, 2015). The emergence of recombinant technologies has revolutionized the selection, humanization, and development of therapeutic antibodies and permitted the strategic design of antibody-based elements for specificity and diversity. A number of genetically engineered mAbs have been used for the treatment of numerous infectious diseases (Hudson and Souriau, 2003).

Ebola virus disease (EVD) is a severe, often fatal, zoonotic infection documented with most recent widespread outbreak in west Africa. It is caused by a virus of the Filoviridae family (genus Ebolavirus). The disease is transmitted from human to human via contact with body fluids of the patients, the incubation period is 1–21 days and case fatality rates range from 30 to 90% (Beeching et al., 2014). A mAb named ZMapp is manufactured in the tobacco plant *Nicotiana benthamiana* to treat EVD patients and to improve public health. It contains a cocktail of different mAbs (MB-003, ZMab, c2G4, and c4G7) that work to avert the spread of the disease within the body (Qiu et al., 2014). A recent study investigated the treatment of EVD patient with ZMapp, a buffy coat transfusion from an Ebola survivor, and the broad-spectrum antiviral GS-5734. The patient is a first neonate documented to have successfully survived EVD (Dörnemann et al., 2017).

Therapeutic antibodies have been generated to fight off several infectious cancers. Cancer is a group of diseases that involve abnormal cell growth and proliferation caused by alterations, or mutations, in the genetic material of the cells. The cell outgrowths or lumps may be benign or metastatic (Marusyk and Polyak, 2010). Cancer outpaces the immune system, avoids detection, or blocks immune system activity. Thus, numerous therapeutic antibodies such as necitumumab, ramucirumab, GRN1005, ganitumab, cixutumumab MM-121, BIIB 033, mapatumumab, trebananib, BHQ880, and carlumab have been investigated for the successful treatment of human cancers. The malignancies include NSCLC, metastatic gastric adenocarcinoma, brain metastasis, breast, ovarian, peritoneal, fallopian tube, prostate, pancreatic, and colorectal cancers, melanoma of the eye and liver, non-Hodgkin lymphoma, and multiple myeloma (Dantas-Barbosa et al., 2012; Nixon et al., 2014).

Similarly, engineered antibodies have been used for the treatment of various types of arthritis. Arthritis (from Greek word “joint”) is a chronic multifactorial disease in which immune system attacks and starts degrading the body's joints (Persidis, 1999). Therapeutic antibodies such as adalimumab, mavrilimumab, and GSK3196165 have been developed for the treatment of rheumatoid arthritis, juvenile idiopathic arthritis, psoriatic arthritis, and ankylosing spondylitis (Dantas-Barbosa et al., 2012; Nixon et al., 2014).

Therapeutic antibodies developed for the treatment of other infectious diseases include prophylaxis, anthrax, autoantibody-positive, lupus, angioedema, immune thrombocytopenic purpura, macular degeneration, hemophilia A, psoriasis, alzheimer, muscle loss and weakness, optic neuritis, ulcerative colitis, pulmonary fibrosis, and asthma (Dantas-Barbosa et al., 2012; Nixon et al., 2014).

Prognosis of a disease is a complex process that involve deduction from several observable symptoms and the choice of treatment based on therapeutic effectiveness. Antibodies, because of their exquisite specificity, and mAbs in general exhibit greater specificity. Therefore, they are used widely in a variety of assay formats, in the diagnosis and treatment of infectious diseases (Zola and Thomson, 2005). Moreover, they show rapid identification of new or rare infectious agents that is an important public health measure. Shortly, development of novel mAbs are helping to monitor and lessen the likelihoods of epidemics and other disease threats imposed on human health by prevalent infectious agents (Zola et al., 2013).

## Conclusions

In the past few decades, antibodies have been developed by using conventional techniques such as hybridomas with significant applications in therapeutics. However, more recently, there have been advancements in recombinant technologies that have enhanced the generation of efficient immunoglobulins and their fragments. Antibody engineering offers rapid, cost-effective, and efficient biomedical tools for the discovery of high-affinity peptides/proteins, investigation of protein-protein interactions, receptor binding, and epitope identification. This powerful technology is used in a variety of systems to approach different questions from a background of cell biology and biotechnology. Moreover, it is used for the production of different types of engineered antibodies against any target molecule or highly unique conserved antigens. Additionally, it is facilitating in diagnosis and treatment of infections to improve human health. Antibody engineering possesses broad spectrum of biological, biotechnological, medical, and antibody applications for the development of novel therapeutics in various disease fields. Numerous novel therapeutic drugs have been developed by *in vitro* screening and selection techniques based on several high throughput immune effector functions, engineered antibodies, and high-affinity antibody fragments. This review has comprehensively described hybridoma technology, advances in antibody engineering techniques, engineered antibodies, antibody fragments, display technologies, and applications of antibodies. In conclusion, the study will help in understanding the significance of antibody fabrication approaches with extensive uses in molecular, immunological, diagnostic, imaging, biomedical, and biotechnological fields, pursuing a healthier future for humans.

## Author contributions

AS, RW, SL, and SW designed the study. AS and SW wrote the paper and all the authors approved its submission.

### Conflict of interest statement

The authors declare that the research was conducted in the absence of any commercial or financial relationships that could be construed as a potential conflict of interest.
